# Family C G-Protein-Coupled Receptors in Alzheimer’s Disease and Therapeutic Implications

**DOI:** 10.3389/fphar.2019.01282

**Published:** 2019-10-28

**Authors:** Ilaria Dal Prà, Ubaldo Armato, Anna Chiarini

**Affiliations:** Human Histology and Embryology Unit, University of Verona Medical School, Verona, Italy

**Keywords:** Alzheimer’s disease, G-protein-coupled receptors, amyloid-beta, calcium-sensing receptor, GABA_B_ receptors, metabotropic glutamate receptors

## Abstract

Alzheimer’s disease (AD), particularly its sporadic or late-onset form (SAD/LOAD), is the most prevalent (96–98% of cases) neurodegenerative dementia in aged people. AD’s neuropathology hallmarks are intrabrain accumulation of amyloid-β peptides (Aβs) and of hyperphosphorylated Tau (p-Tau) proteins, diffuse neuroinflammation, and progressive death of neurons and oligodendrocytes. Mounting evidences suggest that family C G-protein-coupled receptors (GPCRs), which include γ-aminobutyric acid B receptors (GABA_B_Rs), metabotropic glutamate receptors (mGluR1-8), and the calcium-sensing receptor (CaSR), are involved in many neurotransmitter systems that dysfunction in AD. This review updates the available knowledge about the roles of GPCRs, particularly but not exclusively those expressed by brain astrocytes, in SAD/LOAD onset and progression, taking stock of their respective mechanisms of action and of their potential as anti-AD therapeutic targets. In particular, GABA_B_Rs prevent Aβs synthesis and neuronal hyperexcitability and group I mGluRs play important pathogenetic roles in transgenic AD-model animals. Moreover, the specific binding of Aβs to the CaSRs of human cortical astrocytes and neurons cultured *in vitro* engenders a pathological signaling that crucially promotes the surplus synthesis and release of Aβs and hyperphosphorylated Tau proteins, and also of nitric oxide, vascular endothelial growth factor-A, and proinflammatory agents. Concurrently, Aβs•CaSR signaling hinders the release of soluble (s)APP-α peptide, a neurotrophic agent and GABA_B_R1a agonist. Altogether these effects progressively kill human cortical neurons *in vitro* and likely also *in vivo*. Several CaSR’s negative allosteric modulators suppress all the noxious effects elicited by Aβs•CaSR signaling in human cortical astrocytes and neurons thus safeguarding neurons’ viability *in vitro* and raising hopes about their potential therapeutic benefits in AD patients. Further basic and clinical investigations on these hot topics are needed taking always heed that activation of the several brain family C GPCRs may elicit divergent upshots according to the models studied.

## Introduction

Alzheimer’s disease (AD), particularly its sporadic or late-onset form (SAD/LOAD), is by far the most prevalent cause of senile dementia in humans (Alzheimer’s Association, 2018). Typically, multiple neurotoxic factors accumulate in the AD brain, such as soluble amyloid-β oligomers (sAβ-os) and insoluble Aβ fibrils (fAβs), the latter aggregating into senile plaques (Gouras et al., 2015); hyperphosphorylated soluble Tau oligomers (p-Tau-os) that collect into insoluble neurofibrillary tangles (NFTs) (Bloom, 2014); overproduced reactive oxygen species (ROS) (Butterfield and Boyd-Kimball, 2018); nitric oxide (NO); vascular endothelial growth factor-A (VEGF-A), and proinflammatory agents (Dal Prà et al., 2014a; Chiarini et al., 2016). Altogether, these neurotoxins cause a spreading neuroinflammation, progressive synaptic losses, and cortical human neurons and oligodendrocytes deaths with the consequent breaking up of neural circuits. The clinical counterparts of AD neuropathology are steadily worsening losses of memories and cognitive abilities, which inexorably lead to patients’ demise (Dal Prà et al., 2015a; Dal Prà et al., 2015b; Calsolaro and Edison, 2016).

**Figure 1:**
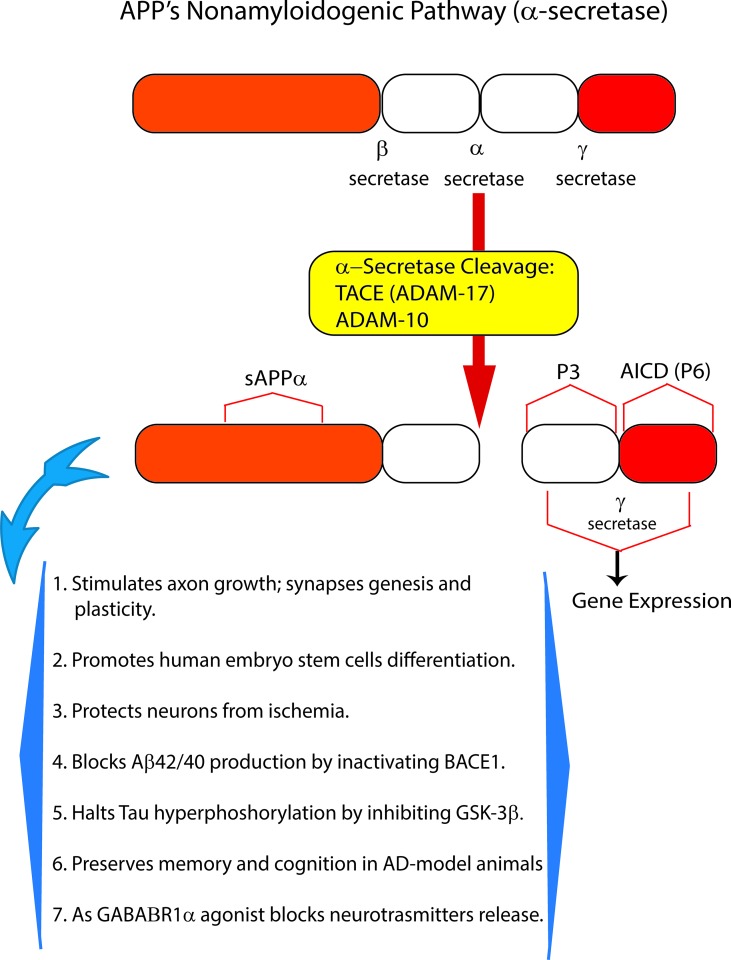
The *nonamyloidogenic processing* (*NAP)* of amyloid precursor protein (APP) holoprotein. By itself, APP holoprotein is not neurotoxic and is cleaved at three different locations by α- or β- and/or γ-secretase. Proteolytic cleavage by α-secretase represents the *NAP* of membrane-inserted APP holoprotein. *NAP* occurs just within the amino acid sequence of Aβ_42_, whose synthesis it consequently obstructs. Thus, α-secretase activity (mostly due to ADAM10) sheds from APP holoprotein the soluble (s)APP-α peptide, whose multiple neurotrophic and neuroprotective effects are summarized in this figure. Recent evidence indicates that as a GABA_B_1aR agonist sAPP-α also constitutively moderates neuronal excitability thus preventing neurons’ harm. In summary, APP holoprotein’s *NAP* hinders the development of AD and preserves neuronal viability, trophism, and function.

**Figure 2:**
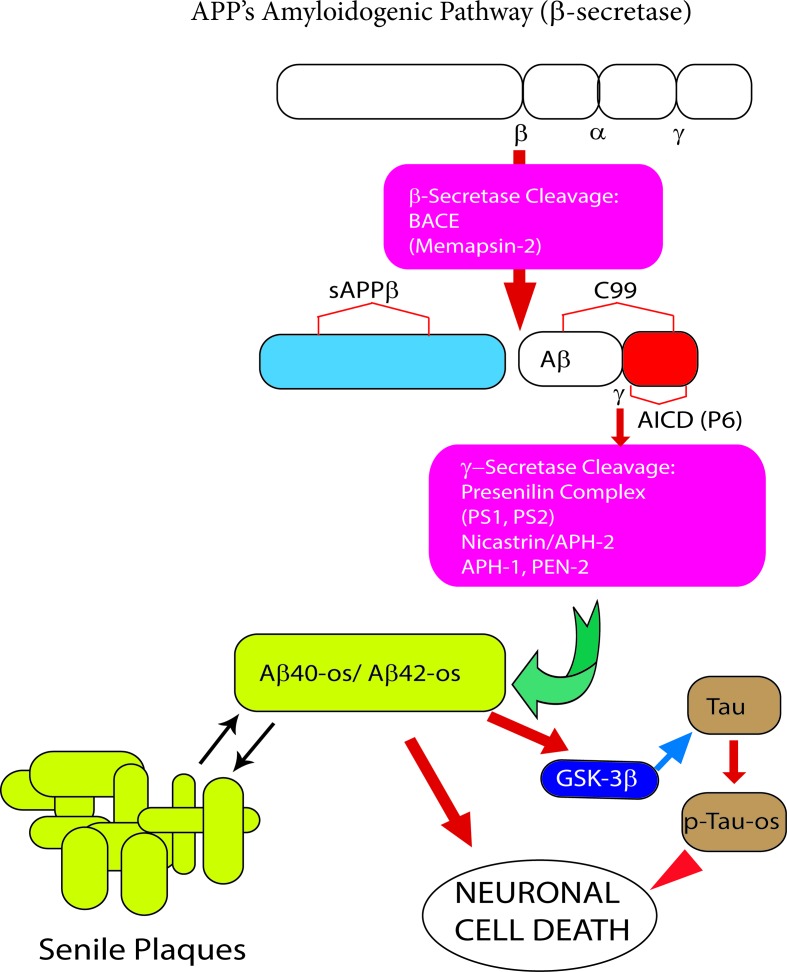
The *amyloidogenic processing (AP)* of amyloid precursor protein (APP) holoprotein. In this pathway β-secretase/BACE1 and γ-secretase sequentially cleave APP holoprotein yielding several Aβ peptide isoforms. The two most prevalent Aβ isoforms are the 40- and 42-amino acid-long residues, the length of which is determined by the cleavage site of the γ-secretase. Under physiological conditions the synthesis of monomeric neurotrophic Aβ peptides is very limited. However, when over produced Aβ peptide monomers end up aggregating first into soluble oligomers (Aβ-os), the first Alzheimer’s disease (AD) drivers, next into insoluble fibrils, and eventually into senile plaques. The latter can both take up and release the neurotoxic Aβ-os. The Aβ_42_ isoform is the main component of senile plaques as is it highly prone to oligomeric and polymeric (fibrillar) aggregation. The Aβ-os interact with several nerve cell membrane receptors, including the calcium-sensing receptor (CaSR). Notably, CaSR-bound Aβ-os trigger a complex set of intracellular signals that promote the development and progression of AD neuropathology (see **Figure 3** for further details).

Amyloid precursor protein (APP), a multifunctional protein widely expressed in the central nervous system (CNS), represents the source of the neurotoxic sAβ-os and fAβs that progressively accumulate in AD brains. Transmembrane APP holoprotein can undergo alternative enzymatic handling: (i) *nonamyloidogenic processing* (*NAP*) by α-secretases that leads to the production of the soluble (s)APP-α while obstructing Aβs synthesis (Chiarini et al., 2017b; Rice et al., 2019) (**Figure 1**); and (ii) *amyloidogenic processing* (*AP*) by β-secretase (BACE1) and γ-secretase liberating Aβs (**Figure 2**). Notably, sAPP-α’s physiological roles are multifaceted, and to-date only partly understood. The available evidence reveals that sAPP-α promotes the neural differentiation of human embryo stem cells (Freude et al., 2011) and protects hippocampal neurons from the harm due to ischemia (Smith-Swintosky et al., 1994), glucose deficiency (Furukawa et al., 1996), brain trauma, and excitotoxicity (Mattson et al., 1993; Goodman and Mattson, 1994). In addition, sAPP-α complexes with and inhibits the activity of BACE1/β-secretase protein thus hindering any excess production of toxic Aβ_42_/Aβ_42_-os (Stein and Johnson, 2003; Obregon et al., 2012; Peters-Libeu et al., 2015). Moreover, sAPP-α stimulates axonal outgrowth (Ohsawa et al., 1997), synaptogenesis, and synaptic plasticity (Hick et al., 2015; Habib et al., 2016). Remarkably, sAPP-α also curbs the activity of glycogen synthase kinase (GSK)-3β and the hyperphosphorylation and overrelease of neurotoxic p-Tau/p-Tau-os, the main components of NFTs (Deng et al., 2015). And an increased activity of GSK-3β has been linked to hyperphosphorylation of Tau in the brains of AD patients. Typically, in AD Tau is phosphorylated at over 30 serine/threonine residues by various protein kinases, including GSK-3β (Pei et al., 1999). The D1 and D6a domains of sAPP-α are the locations of its neuroprotective and neurotrophic activities since they stimulate axons outgrowth when added as separate fragments to *in vitro* hippocampal neurons (Jin et al., 1994; Qiu et al., 1995; Ohsawa et al., 1997). In keeping with such findings, sAPP-α upholds cognition and memory integrity in animal models of physiological aging and of AD (Roch et al., 1994; Meziane et al., 1998; Bour et al., 2004; Ring et al., 2007; Corrigan et al., 2012; Xiong et al., 2016) (**Figure 1**).

SAD/LOAD, which comprises ∼98–96% of the cases, starts from neuronal nests in the layer II of the lateral entorhinal cortex (LEC) in the temporal lobe (Khan et al., 2014) where small ischemic areas may occur in aged subjects (Ishimaru et al., 1996). Thence, in the course of 20–40 years (*asymptomatic stage*) SAD/LOAD silently spreads to wider and wider upper cerebral cortex areas, particularly to those involved in storage and retrieval of memories and in handling complex cognitive activities (Khan et al., 2014). When the unremitting attrition depletes the cortical human neurons’ functional reserve, SAD/LOAD’s first clinical symptoms start manifesting as amnesias. This marks the onset of the *amnestic minor cognitive impairment* or *aMCI stage* that lasts 3–5 years while its symptoms progressively worsen. Eventually, the *full symptomatic stage* takes over, whose exacerbating symptoms include permanent losses of short-term (first) and long-term (later) memories, changes in personality and behavior, loss of the several language-related abilities, failure to cope with daily tasks and needs, motor problems, cognitive shortfalls, dementia, and eventually death. However, it is still hard to diagnose the earliest asymptomatic stage of AD because specific biomarkers are few and the highly neurotoxic, synapse-destroying sAβ_42_-os are hardly detectable when senile plaques and NFTs are still absent (Selkoe, 2008a; Selkoe, 2008b; Ferreira and Klein, 2011; Klein, 2013; Dal Prà et al., 2015a). Even so, the ghostly sAβ_42_-os eventually cause a noticeable accumulation of Aβ_42_ as fibrils and senile plaques, and of p-Tau-os as NFTs (Medeiros et al., 2013). Presently, the diagnosis of SAD/LOAD is based upon detecting brain deposits of insoluble Aβs (senile plaques) *via* PET imaging and specific changes in Aβ_42_/Aβ_40_ and Tau/p-Tau ratios values in the cerebrospinal fluid (CSF), which are deemed to be pathognomonic (McKhann 2011). PET imaging can also detect the brain accumulation of NFTs (Hall et al., 2017). The quest of blood biomarkers of AD is still ongoing with some preliminary promising results (Nabers et al., 2018; Nakamura et al., 2018; Palmqvist et al., 2019).

Presently, no drug therapy modifies or mitigates AD’s relentless course (Jessen et al., 2014). This unsatisfactory situation still lingers because of various reasons. First, SAD/LOAD pathogenesis remains unclear and, hence, an open to speculation topic. Second, animal models closely mirroring human SAD/LOAD are as yet not available (Ameen-Ali et al., 2017). Transgenic (*tg*) animal (mostly rodent) AD-models only partially and imperfectly emulate the early-onset familial (EOF)AD variety, which comprises at most 2–4% of AD cases. It is well established that EOFAD results from mutations in the amyloid precursor protein (*APP*) or presenilin1 (*PSEN1*) or presenilin2 (*PSEN2*) genes. These mutations drive a constitutive, diffuse intrabrain overproduction and overload of sAβ-os, insoluble Aβ fibrils, and hence Aβ-heaped senile plaques and concurrently of p-Tau-os and NFTs. Conversely, no genetic mutations underlie SAD/LOAD pathogenesis, although *APOE* (Huang and Mahley, 2014) and *TREM2* (Gao et al., 2017) gene variants could increase AD proclivity. While Aβ-os appear as the first main AD drivers, in such *tg* AD-model animals p-Tau accumulates as NFTs later and only when a mutated *MAPT* transgene is inserted too (Oddo et al., 2003; Cohen et al., 2013). *Third*, AD-model rodent brains substantially differ under several cytological, structural, and functional standpoints from the human brain. Some differences are obvious, such as brain size and weight, the cerebral cortex limited extent, the largely prevailing primitive olfactory cortex, and so on. Other differences are more subtle, but still exceedingly important, as they critically regard a genetic homology of only 80% and structural and functional features of cortical neurons and neuroglial cells--e.g. the total absence of some human cortical astroglial subtypes from rodent brain cortices--and the dissimilar extension of the astrocytes’ domains, and the unlike reactions (e.g. Ca^2+^ fluxes) of each neural cell type once exposed to AD-driving neurotoxins. As a revealing example, in *tg* AD-model animals, the cortical astrocytes die sooner than neurons, whereas cortical neurons die earlier than astrocytes in human AD brains. Altogether, such a complex set of divergences has suggested that *tg* rodent AD-model animals may not be the ultimate means to identify therapeutic approaches benefiting human AD patients (Ransohoff, 2018). Hitherto, all drugs that resulted advantageous when given to *tg* AD-model animals have failed to act as beneficial therapeutics in human AD patients (Cummings, 2017). It should be noted that this is a currently recognized general problem affecting the quest for and successful trial of novel drugs preclinically tested in animal models of other human diseases besides AD (Gabrielczyk, 2019). Unquestionably, novel rodent and non-human primate (NHP) AD models are under development (Podlisny et al., 1991; Sasaguri et al., 2017) but their potential worth for human AD therapeutic research remains to be assessed.

So, are there acceptable alternatives to AD-model animals? Given the just mentioned species-related differences, one should take stock of untransformed human neural cells making up “*preclinical AD models in Petri dishes*”. An example of this kind may be neural cells differentiated from human induced pluripotent stem cells (iPSCs) isolated from normal subjects and/or from EOFAD and SAD/LOAD patients and set into 2D or 3D cultures (Kim et al., 2015). Human iPSC models are easier to handle than NHP models and may also be integrated into mouse AD models (Espuny-Camacho et al., 2017).

Still another suitable preclinical human AD model *in vitro* consists of untransformed cortical adult human astrocytes and/or neurons. Human astrocytes from the temporal lobe cerebral cortex exhibit a stable (“*locked-in*”) differentiated phenotype and give reproducible responses when exposed to fAβs and/or sAβs. The experimental exploitation of the latter cells has revealed that exogenous Aβs specifically bind the calcium-sensing receptor (CaSR) (Dal Prà et al., 2014a; Dal Prà et al., 2014b; Dal Prà et al., 2015b), a member of family C G-protein coupled receptors (GPCR), and activate a pathological signaling that could drive human LOAD/SAD onset and progression and also worsen EOFAD’s course. These findings have clearly pointed out to a class of therapeutic agents, the CaSR negative allosteric modulators (NAMs), which effectively block all such AD’s pathogenetic mechanisms in untransformed cortical human neurons and astrocytes *in vitro* and could stop the progression of AD neuropathology in the patients (Armato et al., 2013; Chiarini et al., 2017a; Chiarini et al., 2017b).

Family C GPCRs also include the metabotropic glutamate (mGlu) and GABA_A/B_ receptors (Bräuner-Osborne et al., 2007; Urwyler, 2011). Results of studies in animal model suggest that mGlu-Rs and GABA-Rs might also be involved in AD pathophysiology because AD concurs with alterations of glutamatergic transmission (Caraci et al., 2018). Therefore, we discuss here the roles of family C GPCRs in AD progression and hence their potential relevance to human AD therapy.

## Family C G-Protein-Coupled Receptors

In general, GPCRs are among the most numerous groups of transmembrane proteins of the mammalian genome. To date, about 800 of these proteins have been identified in humans (Fredriksson et al., 2003). The relevance of their manifold functions has made them therapeutically attractive as shown by the fact that they are the targets of ∼34% of United States Food and Drug Administration-approved drugs (Hauser et al, 2017). Currently, GPCRs are distinguished into six classes (families A–F) (**Table 1**) based upon amino acid sequence homologies, elected signal transduction pathways, and pharmacological outlines.

**Table 1:** G-Protein-coupled receptors (GPCRs) Families.

Family A	rhodopsin-like receptors
Family B	secretin receptors
Family C	γ-aminobutyric acid B (GABA_B_) receptors; metabotropic glutamate receptors (mGluR1-8); calcium-sensing receptor CaSR; taste receptors 1-3; *V2 pheromone receptors; *GPRC6A receptor;**various orphan receptors.**
Family D	fungal mating pheromone receptors
Family E	cyclic AMP receptors
Family F	frizzled/smoothened receptors

*These receptors abound in rodents but are absent from humans

(Niswender and Conn 2010; Alexander et al., 2017).

**Not considered in this review.

Details concerning the structure of family C GPCRs are known for the mGluRs (Kunishima et al., 2000; Tsuchiya et al., 2002; Muto et al., 2007; Doré et al., 2014; Wu et al., 2014), GABA_B_R (Geng et al., 2013), and CaSR (Gama et al., 2001; Geng et al., 2016) extracellular domains (ECDs), and for the mGluRs and CaSR transmembrane domains (Doré et al., 2014). Family C GPCRs share a common general structure characterized by a huge bilobed N-terminal extracellular domain (ECD) or “Venus Flytrap” (VFT) (Fredriksson et al., 2003; Lagerström and Schlöth, 2008; Rosenbaum et al., 2009). A cysteine-rich region (CR) links the ECD/VFT to the 7TM domain including seven transmembrane helical hydrophobic regions (TM1–TM7) connected extracellularly by three loops (ECL1–ECL3) and intracellularly by three loops (ICL1–ICL3). The CR domain is extant in all family C GPCRs save for GABA_B_Rs. Finally, the 7TM domain is linked to the intracellular C-terminal domain (ICD), whose tail interacts with G proteins to activate downstream signaling pathways.

Family C GPCRs function as mandatory dimers (El Moustaine et al., 2012) joined by a disulfide bond topping the two VFTs. GPCRs can be formed into homodimers or into heterodimers with other members of the same group or family (Goudet et al., 2005; Doumazane et al., 2011; Kammermeier, 2012; Sevastyanova and Kammermeier, 2014) or with extraneous GPCRs (Ciruela et al., 2001; Gama et al., 2001; Cabello et al., 2009; Kniazeff et al., 2011). The orthosteric ligands bind the pockets placed in the slit between the two VFT’s lobes causing the active closure of both slits (closed-closed conformation) or of only one slit (open-closed conformation) (Parnot and Kobilka, 2004). Conformational changes inside the VFT domains are conveyed through the cysteine-rich and 7TM regions to the ICD domain to regulate G-proteins binding and activate intracellular signals (Rondard et al., 2006).

The family C GPCRs subtype specificity of orthosteric agonists and antagonists varies because the amino acid sequence of the VFT binding pocket may or may not be extensively conserved. Group III mGluRs are an example of the former case, as their orthosteric agonists and antagonists are possessed of a mostly unchanging broad-spectrum activity (MacInnes et al., 2004; Austin et al., 2010). To overcome this obstacle to therapeutic applications, an active quest has been and still is going on for drugs that bind on particular amino acid sequences defined as allosteric sites or pockets (e.g. on the ECL1–ECL3 of the 7TM domain) and placed well outside the VFT-inside orthosteric site. A number of allosteric sites located within the 7TM domain were identified by investigations using site-directed mutagenesis and allosteric modulator cocrystal methods (Gregory et al., 2012;Doré et al., 2014; Wu et al., 2014; Christopher et al., 2015). These novel drugs selectively modify the receptor signaling triggered by an orthosteric ligand acting as either positive allosteric modulators or PAMs or negative allosteric modulators or NAMs (Engers and Lindsley, 2013). PAMs favor whereas NAMs hinder the binding affinity/activity (the so-called cooperativity) of orthosteric ligands. Finally, neutral allosteric ligands (NALs) bind their sequence on a receptor but do not change the cooperativity of its orthosteric ligand (Wootten et al., 2013; Christopoulos, 2014). The otherwise steady structural conformation of orthosteric ligand-bound GPCRs undergoes transient changes, which impact on the interactions with G proteins or other transducers, when the receptors also bind allosteric modulators (Canals et al., 2011). Lipophilic PAMs and NAMs cross the blood–brain barrier (BBB) (Ritzén et al., 2005). However, extremely lipophilic PAMs and NAMs may exhibit a lesser ability to reach the neural cells while their unwanted side effects may become stronger (Goudet et al., 2012). Present-day methods used to evaluate allosteric interactions, i.e. functional and radio-ligand binding assays, need proper probes to reveal the receptor’s response and fully disclose the allosteric ligand’s properties (Price et al., 2005; Hellyer et al., 2018). The affinity and effectiveness of allosteric modulators can be quantified *via* the allosterism’s operational model that associates the allosteric ternary complex model (ATCM) with Black–Leff’s operational model of pharmacological agonism (Black and Leff, 1983; Ehlert, 1988; Leach et al., 2007; May et al., 2007). The latter allows to quantify the modulator’s effectiveness and its impact on orthosteric agonist affinity vs. efficacy (Black and Leff, 1983; Leach et al., 2007; Keov et al., 2011; Gregory et al., 2012). Classically, NAMs and PAMs are effective only when the natural orthosteric ligands (or probes) are present (Conn et al., 2009). At times, surrogate orthosteric probes are required for functional assays. Probes of different chemical nature may affect the cooperativity in opposite directions or leave it unchanged (Koole et al., 2010; Valant et al., 2012; Sengmany et al., 2017). Remarkably, allosteric modulators evoke saturable effects, i.e. no further activity obtains when they reach saturating doses (May et al., 2007; Klein, 2013). Specific PAMs and NAMs may or sometimes may not elicit the internalization of their receptors, and hence may or may not desensitize the cells to corresponding orthosteric ligands (Conn et al., 2009). Some PAMs and NAMs are of a mixed type acting as both orthosteric agonists and PAMs, while others act as both agonists for one receptor subtype and as antagonists for another receptor subtype. Preferred PAMs or NAMs should not permanently activate or inactivate the involved receptors because this could elicit harmful effects (Célanire and Campo, 2012). Clearly, receptor subtype-specific PAMs and NAMs are indispensable tools for basic and preclinical pharmacological research and, when beneficial, might be transformed into drugs apt for clinical trials. For example, one chemokine receptor-5 NAM, i.e. Maraviroc, successfully reached the clinical use to treat late-stage HIV disease (Dorr et al., 2005).

Here, a cautionary note is in order about the interspecies translatability of experimental results related to family C GPCRs. In fact, brain locations of the same family C GPCRs can significant vary between animal species (e.g. mouse vs. rat) and between animals and humans. Such species-related divergences could explain the inconsistent observations one may make when experimenting with more than one animal species. This is a further drawback to translating the beneficial effects evoked by orthosteric agonists/antagonists or allosteric PAMs/NAMs in animal models of diseases into beneficial therapeutic upshots in disease-matching human patients. Moreover, one should not overlook another caveat concerning the extrapolation to postnatal life of results gained by administering PAMs or NAMs to animal embryo-fetal cellular models. In fact, during development mGluRs expression undergoes divergent changes in distinct types of cells. Finally, on a positive note, the combined administration of orthosteric agonists or antagonists with NAMs or PAMs can provide additive or synergistic neuroprotection, which might have future therapeutic applications (Vernon et al., 2005; Bennouar et al., 2013).

## GaBA_B_Rs, APP, Aβs, and AD

There are two classes of GABARs, i.e. GABA_A_Rs and GABA_B_Rs. While GABA_A_Rs are fast-acting ionotropic receptors functioning as ligand-gated ion channels, GABA_B_Rs are metabotropic family C GPCRs, whose structure comprises the subunits R1a, R1b, and R2 (Chang and Shoback, 2004). Receptor dimerization or oligomerization is obligatory for GABARs as it impacts on function. Only GABA_B_ R1a/R2 or R1b/R2 heterodimers do reach the cell surface, bind GABA to R1 subunit, and activate G-protein-mediated intracellular signals *via* R2 subunit (White et al., 1998; Marshall et al., 1999; Margeta-Mitrovic et al., 2001). Of note, GABA_B_R1 and R2 subunits form heterodimers also with the CaSR (this topic will be further discussed below). Two “*sushi domains* (SDs)” abut from the N-terminus of GABA_B_R1a VFT, which are required for the receptor’s trafficking to the cell surface and for its presynaptic inhibitory activity (Gassmann and Bettler, 2012; Hannan et al., 2012). Adaptor proteins link the GABA_B_R1a SD1 domains to axoplasmic kinesin-1 motors. Recently, proteomic methods permitted to identify three adaptor proteins playing this role, i.e. APP, adherence-junction associated protein 1 (AJAP-1), and PILRα-associated neural protein (PIANP). Thus, axonal trafficking cargo vesicles carry at least three distinct types of GABA_B_R1a/adaptor protein/kinesin-1 complexes. It is noteworthy that the formation of any of such GABA_B_R1a/APP/kinesin-1 complexes obstructs the amyloidogenic processing (*AP*) of the involved APP molecules into Aβ_42_/Aβ_42_-os, the AD main drivers. This could be a novel AD-preventing mechanism involving GABA_B_R1a axonal trafficking (Dinamarca et al., 2019).

Neurons express GABA_B_Rs on both presynaptic and postsynaptic membranes. Neuronal activity controls GABA_B_Rs presynaptic expression (Guetg et al., 2010; Terunuma et al., 2010; Orts-Del’Immagine and Pugh, 2018). Conversely, model animals of AD (Chu et al., 1987; Iwakiri et al., 2005) and of various other diseases, like chronic epilepsy (Thompson et al., 2006–2007), fragile X syndrome (Kang et al., 2017), and Parkinson disease (Borgkvist et al., 2015), downgrade GABA_B_Rs expression and inhibitory function causing neuronal hyperexcitability. Human astrocytes express both GABA_A_Rs and GABA_B_Rs and constitutively synthesize and release GABA, therefore being GABAergic cells. GABA release from human astrocytes is dose-dependently increased by glutamate or by NMDAR coagonists like D-serine and glycine. Conversely, inhibitors of kainic acid receptors and of NMDARs decrease GABA release from human astrocytes. Interestingly, the administration of exogenous GABA suppresses the proinflammatory responses of activated astrocytes and microglia to noxious stimuli (Lee et al., 2011a; Lee et al., 2011b).

The GABA_B_Rs classical ligand is γ-aminobutyric acid (GABA), which is the chief inhibitory neurotransmitter of the mature central nervous system (CNS) of vertebrates. GABA-releasing (GABAergic) neurons are ubiquitous in the CNS. Nearly half of all CNS synapses express some GABA_B_Rs and thus respond to GABA (Li et al., 2016). The signaling activated by the GABA•GABA_B_R complexes inhibits the release of neurotransmitters from the targeted postsynaptic neurons (Benes and Berretta, 2001; Bettler et al., 2004). But the signaling activated by the presynaptic GABA•GABA_B_R complexes of the GABAergic neurons blocks their further GABA release thus acting as a physiological self-controlling mechanism exerting an indirect excitatory effect on postsynaptic neurons. An astrocyte → neurons metabolic cycle upkeeps the neuronal stores of GABA (Losi et al., 2014; Mederos et al., 2018). The released GABA moieties are taken up by both pre- and postsynaptic neurons and by the astrocytes enveloping tripartite synapses. In their mitochondria, the astrocytes convert the uptaken GABA into glutamine and forward the glutamine to adjoining neurons. In presynaptic neurons, two sequentially acting enzymes synthesize GABA from glutamine: first, glutaminase, which converts glutamine into glutamate; and second, glutamic acid decarboxylase (GAD), which transforms glutamate into GABA. Next, the vesicular transporter of GABA (VGAT) transfers it into cargo/synaptic vesicles, which release GABA into the synaptic cleft after the neuron’s membrane depolarization. GABA signaling regulates several physiological aspects of CNS activity, like neurogenesis, neuronal development, sexual maturation, circadian rhythms, motor functions, learning, and memory (Petroff, 2002). GABAergic interneurons are the chief inhibitory neurons in the CNS. Abundant reports in the literature stress how dysfunctions of the activity of GABAergic interneurons disrupt the glutamatergic excitatory/GABAergic inhibitory (E/I) balance in neural circuits of the cerebral cortex, hippocampus, and subcortical structures (e.g. amygdala) causing declining cognitive capabilities and worsening memory losses. Therefore, it is generally held that E/I imbalance significantly impacts on the pathogenesis of AD (Govindpani et al., 2017; Villette and Dutar, 2017) and of various other CNS disorders, such as major depression (Fee et al., 2017), schizophrenia, autism’s spectrum, bipolar disorder (Benes and Berretta, 2001; Lehmann et al., 2012; Gao and Penzes, 2015; Xu and Wong, 2018), and anxiety (Babaev et al., 2018).

Unfortunately, the results of postmortem studies on AD patients did not throw much light onto GABA_B_Rs role(s) in AD and this for good reasons--one being the wide-ranging variability of the patients’ terminal lifetime. Different alterations of biological parameters--such as expression of RNA, proteins, and enzymes--can be induced by co-morbidities, medications, aging, and leading death causes and are all directly linked to the duration and severity of the full-blown or symptomatic AD phase (Govindpani et al., 2017). Therefore, it is not surprising that divergent findings abound concerning GABA_B_Rs’ possible role(s) in AD. Thus, recent postmortem studies of human brains and of *tg* AD-model animals reported that GABAergic neurons and GABA_B_Rs were unaffected by AD neuropathology (Li et al., 2016). Conversely, earlier studies had conveyed the opinion that GABA_B_Rs signaling undergoes profound changes in AD (Takahashi et al., 2010). Significantly lowered GABA levels were detected in the temporal cortex of AD patients (Gueli and Taibi, 2013) and in cerebrospinal fluid (CSF) samples from both AD patients and the cognitively normal elderly (Grouselle et al., 1998). Conversely, raised GABA levels turned up in the hippocampus and CSF samples of AD patients and were ascribed to the impairment of synaptic plasticity (Jo et al., 2014). These studies also noted that the reactive astrocytes surrounding Aβs senile plaques overproduced GABA *via* monoamine oxidase-B (MAO-B) activity and abnormally released it through the bestrophin-1 channels. Under physiological conditions, bestrophin-1 channels are mostly localized at the microdomains of hippocampal astrocytes nearby glutamatergic synapses and mediate glutamate release. A switch from the glutamate-releasing normal astrocyte to the reactive astrocyte releasing GABA *via* bestrophin-1 channels is a common phenomenon occurring in various pathological conditions coupled with astrogliosis, such as traumatic brain injury, neuroinflammation, neurodegeneration, and hypoxic and ischemic insults. In AD, bestrophin-1 channels are redistributed to the soma and the processes of hippocampal reactive GABA-containing astrocytes. Bestrophin-1 channels-mediated GABA release from reactive astrocytes hinders synaptic plasticity and transmission and spatial memory by reducing dentate granule cell excitability (Oh and Lee, 2017). It was claimed that suppressing GABA overproduction by monoamine oxidase-B (MAO-B) or GABA overrelease through bestrophin 1 channels from the dentate gyrus reactive astrocytes fully restored learning and memory in AD-model mice (Jo et al., 2014). However, the long-term administration of selegiline, an irreversible MAO-B inhibitor, did not improve AD neuropathology in a clinical trial (Park et al., 2019). To explain this unforeseen upshot, the authors suggested that multiple factors, like age, sex, and differences in brain regions could impact on the GABA release from astrocytes and neurons and should not be ignored when planning therapeutic drug attempts. Indeed, different brain regions of Tg2576 (human APP695 plus the Swedish double mutation K670N, M671L) AD-model mice released dissimilar amounts of GABA in relation to their actual age and sex. Cortical GABA levels were higher in older than 6 months female than male mice; however, at a more advanced age, this difference vanished in the parietal cortex but became more pronounced in the prefrontal cortex. Moreover, at 12–18 months of age, hippocampal levels of GABA were lower in female than in male mice. Altogether, these data revealed that with advancing age the functional disruption of GABA signaling turns out to be more intense in AD-model female mice (Hsiao et al., 1996). Conversely, under or up to 9 months of age, hippocampal GABA levels were higher in female than in male mice, likely because the former enjoyed the protective activity of estrogens (Roy et al., 2018). By extrapolating these data from animals to humans one can infer that a single therapeutic strategy addressing GABA_B_Rs modulation might not be so easily feasible in AD. In fact, any drug inhibiting the GABA_B_Rs residing in one brain region might exacerbate the dysregulation of GABAergic signaling in other brain areas.

Hence, the role(s) of GABA/GABA_B_Rs signaling in the pathogenesis of AD has(ve) hitherto remained unclear if not confusing (Govindpani et al., 2017). However, the recent studies we mention just below performed on wild-type and AD-model animals have thrown some more light on the contribution of GABAergic remodeling to the pathogenesis of both early and late stages of AD.

First, we recall here that the ε4 allele of apolipoprotein E (APOE) is the main known genetic risk factor for LOAD/SAD. Notably, in the brains of aged APOEε4 mice, an attenuation of GABAergic inhibitory inputs on associated excitatory neurons drives a specific neuronal hyperactivity phenotype. Hence, an APOEε4-driven hippocampal neuronal excitatory hyperactivity might be among the causative factors underlying the increased risk of AD among APOEε4 carriers (Nuriel et al., 2017). In addition, several AD-mouse models exhibit an early and marked neuronal hyperactivity in the hippocampus (Busche et al., 2012; Busche et al., 2015). Moreover, functional magnetic resonance imaging (fMRI) studies have revealed that humans with mild cognitive impairment (MCI), as well as presymptomatic carriers of EOFAD mutations show enhanced neuronal activity in the same brain region, the hippocampus (Quiroz et al., 2010; Haberman et al., 2017). Therefore, when the increase in brain activity takes place early in the pathogenetic process it may be rightly considered a driving factor in AD development.

Second, according to a recent report, Aβs, AD’s main drivers, are intensely degraded by endothelin-converting enzyme-2 (ECE-2) and neprilysin (NEP) in the somatostatinergic and parvalbuminergic synapses of GABAergic interneurons residing in the neocortex and hippocampus. These observations support the view that under physiological conditions Aβs may partake in the regulation of interneurons’ inhibitory signaling in AD-relevant brain areas (Pacheco-Quinto et al., 2016). However, it must be stressed here that a reduction of Aβs catabolism at the synapses of these two distinct populations of GABAergic neurons is not the unique GPCRs-mediated mechanism favoring Aβs accumulation in AD brains.

Third, the exciting results of very recent studies have revealed that GABA_B_R1a receptors bind three novel orthosteric agonists besides GABA, i.e. soluble (s)APP-α, sAPP-β, and sAPP-η proteins. The α-, β-, and η-secretases, respectively, shed them from the extracellular domain of APP into the brain environment. Next, each of these peptides can bind GABA_B_R1a receptors and block the release of neurotransmitters from hippocampal presynaptic excitatory axonal terminals thus silencing synaptic transmission. Most interesting, a 17-mer peptide of the ExD flexible portion of sAPP-α, which binds the extracellular Sushi1 domain of the GABA_B_R1a could replicate the squelching effect on neurotransmission brought about by the whole sAPP-α molecule. These results explain, at least in part, the synaptic dysfunction affecting some APP-overexpressing AD-model animals. Moreover, they suggest that this 17-mer peptide could therapeutically counteract the excitatory hyperactivity of neuronal synaptic function brought about by Aβs (Rice et al., 2019; Tang, 2019).

## mGLURs AND AD

The seven-transmembrane-spanning mGluRs physiologically control synaptic transmission and neuronal excitability in the CNS and influence behavioral output processes. These receptors are assigned to three groups according to their G-protein coupling and signal transduction pathways. Group I encompasses mGluR1 and mGluR5; group II includes mGluR2 and mGluR3; and group III embraces mGluR4, mGluR6, mGluR7, and mGluR8. In general, group I receptors are coupled to the phospholipase (PL)C/InsP_3_/Ca^2+^ release cascade, whereas groups II and III receptors are linked up to the adenylyl cyclase/cyclic AMP/PKA release cascade (Niswender and Conn, 2010). Initial studies performed with agonists (or antagonists), which bind the intragroup-shared extracellular orthosteric sites, indicated that activation of group II or group III mGluRs brought about neuroprotection, whereas activation of group I mGluRs elicited either neuroprotection or neurotoxicity according to experimental models and conditions employed (Nicoletti et al., 1999; Bruno et al., 2001). More recent studies using PAMs or NAMs, which are receptor subtype-specific, brought to light a somewhat different picture (see for references: Gregory and Conn, 2015). Indeed, the allosteric modulation of mGluRs is a major area of interest for Basic and Clinical Pharmacology (Stansley and Conn, 2019). In the CNS, mGluRs are involved in the regulation of glutamate uptake, cell proliferation, neurotrophic support, and proinflammatory responses. Accordingly, the potential therapeutic spectrum of mGluRs allosteric modulators embraces AD, and also covers PD, stress, anxiety, autism, depression, and schizophrenia (Stansley and Conn, 2019).

### Group I mGluRs (-1 and -5)

Group I mGluR1 and mGluR5 are expressed at postsynaptic membranes, couple to Gαq, and positively modulate neuronal excitability through the interaction with scaffolding proteins such as Homer or Shank. The consequent activation of phospholipase C leads to an increase in [Ca^2+^]_i_. Activation of group I mGluRs may set off a multiplicity of neurons’ and astrocytes’ signaling pathways variously modulating synaptic plasticity and, likely, synaptic protein synthesis (D’Antoni et al., 2014). These transduction mechanisms form a highly complex network including polyphosphoinositide hydrolysis, mitogen-activated protein kinase/extracellular signal-regulated kinase (MAPK/ERK), phospholipase D, phospholipase A2, phosphoinositide 3-kinase (PI3K), mammalian target of rapamycin (mTOR), and the endocannabinoid 2-arachidonoylglicerol synthesis. Activation of ERK and mTOR by group I mGluRs is especially linked to *de novo* protein synthesis in neurons, a process that underlies long-term changes in activity-dependent synaptic plasticity. Group I mGluRs also enhance postsynaptic excitability thus exacerbating neuronal damage (Nicoletti et al., 1996). It is also noteworthy to recall here that in preclinical studies *antagonists* of mGluR1 and mGluR5 exhibited anxiolytic-like properties just as did *agonists* of group II/III mGluRs (Stachowicz et al., 2007).

By interacting with NMDARs, mGluR1 and mGluR5 regulate neuronal developmental plasticity. The interaction between group I mGluRs and NMDARs is reciprocal (Alagarsamy et al., 1999). Moreover, the expression of these receptors is developmentally regulated (Nicoletti et al., 1986; Schoepp and Johnson, 1989; Minakami et al., 1995; Romano et al., 1996; Casabona et al., 1997). Group I mGluRs are cross-linked with NMDARs through a chain of anchoring proteins (Tu et al., 1999), and their activation amplifies NMDA currents (Aniksztejn et al., 1995; Awad et al., 2000; Pisani et al., 2001; Skeberdis et al., 2001; Heidinger et al., 2002; Kotecha and MacDonald, 2003). In addition, activation of mGluR1 accelerates NMDARs trafficking (Lan et al., 2001). The NMDA component of long-term potentiation (LTP) is abolished in mice lacking mGluR5 (Jia et al., 1998). In cultured neurons and developing brains the interaction between mGluR5 and NMDAR is amplified by EphrinB2 (Calò et al., 2005), a ligand for EphB receptor tyrosine kinases playing a role in activity-dependent synaptic plasticity in the CNS (Slack et al., 2008).

In the developing brain, mGluR5 contributes to the functional maturation of astrocytes since mGluR5 ablation leads to serious deficits in arborization of astroglial processes and in the expression of glutamate transporters (Morel et al., 2014; Verkhratsky and Nedergaard, 2018).

To date, the roles of group I mGluRs in the pathogenesis of AD are poorly understood and the object of controversy.

*In vitro* cultured fetal (E15) Sprague–Dawley rat neurons expressed mGluR1 whereas neonatal astrocytes did not. These findings limited to neurons the investigation of an alleged neuroprotective effect of the mGluR1/R5 *agonist*, 3,5-dihydroxyphenylglycine (DHPG), against Aβ neurotoxicity, which was instead suppressed by the mGluR1 *antagonist* JNJ 16259685 (3,4-dihydro-2H-pyrano(2,3-b)quinolin-7-yl)-(cis-4-methoxy-cyclohexyl)-methanone). Interestingly, estrogen-α receptors (E-αRs) could activate the same neuroprotection against Aβ toxicity and cell survival pathways as mGluR1 did. Indeed, E-αRs and mGluR1 were colocalized in cultured cortical neurons and were interdependent in activating the PI3K/Akt pathway that favors cell survival in pure neuronal cultures (Spampinato et al., 2012).

As regards mGluR5s, they are expressed by both astrocytes and neurons in all the CNS areas, signal through Gq protein (Vanzulli and Butt, 2015), and partake in synaptic plasticity, assembly of neuronal circuitry, and neuronal viability (Ballester-Rosado et al., 2010; Purgert et al., 2014).

Data gained from both *in vitro* and animal models suggest that the synaptic dysfunction of mGluR5s might favor the development of AD (Kumar et al., 2015). mGluR5s are overexpressed by astrocytes as a reactive response induced by stimulation with growth factors (i.e. FGF, EGF, and TGF-β1) or by exposure to soluble Aβ oligomers (Aβ-os) *in vitro* (Casley et al., 2009; Grolla et al., 2013; Lim et al., 2013). Aβ-os exposure also raises the expression of type I InsP3Rs, which are placed downstream from mGluR5, and strengthens Ca^2+^ responses mediated through the mGluR5/InsP_3_R cascade in hippocampal astrocytes (Grolla et al., 2013). Notably, astrocytes surrounding Aβ senile plaques overexpressed mGluR5, which was associated with Ca^2+^ signaling dysregulation and abnormal ATP release in APP_swe_/PS1 transgenic AD-model mice (Shrivastava et al., 2013). Reportedly, Aβ-os exposure caused an excessive clustering and widely reduced diffusion of Aβ-os/mGluR5 complexes on the plasma membrane of *in vitro* rat embryo astrocytes. These effects were coupled with an augmented Ca^2+^ influx altogether damaging synapses (Renner et al., 2010; Shrivastava et al., 2013). Activation of mGluR5s by the allosteric agonist DHPG increased ATP release from Aβ-os-exposed astrocytes, which delayed mGluR5 diffusion in cultures of astrocytes plus/minus neurons *in vitro* (Renner et al., 2010)—an effect mGluR5’s selective antagonist MPEP counteracted thus preventing Aβ-os/mGluR5-driven synaptotoxicity (Shrivastava et al., 2013).

Interestingly, proinflammatory cytokines like interleukin-1β (IL-1β) and tumor necrosis factor-α (TNF-α) downregulated the expression of mGluR5 while upregulating that of mGluR3 in cortical astrocytes isolated from the hSOD1(G93A) rat model of amyotrophic lateral sclerosis (entailing like AD an intense neuroinflammation) and cultured *in vitro*. These findings suggested the existence of a protective antiexcitotoxic adaptive mechanism (Berger et al., 2012). In fact, the mGluR5 selective antagonist MPEP hampered the astrocytes’ secretion of two proinflammatory cytokines, IL-6 and IL-8 (Shah et al., 2012). Therefore, the activation of astrocytes’ mGluR5 advances the release of proinflammatory cytokines, which then downregulate mGluR5 expression. This indicates that under physiological conditions a reciprocal feed-back mechanism controls the expression levels of mGluR5 in astrocytes and in microglia too (Berger et al., 2012). This mechanism might be offset by the Aβ-os-forced overexpression of mGluR5 in AD, thus potentiating the release of toxic amounts of proinflammatory cytokines and glutamate. Next, the latter increases the production/release of p-Tau-os and of NO and the activity of apoptotic caspase-3 (Talantova et al., 2013; Lee et al., 2014).

Another noteworthy study showed that the activation of mGluR5 stimulated the α-secretase-mediated extracellular shedding of neurotrophic and neuroprotective sAPP-α (Sokol et al., 2011), also an agonist of GABA_B_R1a receptors (Rice et al., 2019). But mGluR5 forms complexes with the Homer proteins that interact with and activate NMDARs (Tu et al., 1999; Awad et al., 2000; Attucci et al., 2001; Moutin et al, 2012). Aβ_1-42_-os can bind mGluR5s and enhance their clustering together, causing mGluR5 signaling overactivation, intracellular Ca^2+^ accumulation, impaired calcium homeostasis, and synaptic disruptions (Renner et al., 2010; Zhang et al., 2015b). In greater detail, mGluR5s act as coreceptors for Aβ-os bound to prion protein (PrP^c^). Next PrP^c^ activates the mGluR5, which elicits the loss of synapses through *Fyn* tyrosine kinase activation and eukaryotic elongation factor 2 (eEF2) phosphorylation (Um et al., 2013). *Fyn* phosphorylates NR2A and NR2B subunits of NMDA receptors thus altering the receptors’ intracellular trafficking that is essential for synaptic plasticity. Moreover, interactions between *Fyn* tyrosine kinase and Tau proteins play a role in regulating the synapse function and the postsynaptic toxic signaling pathways driven by Aβ-type excitotoxicity, causing the loss of dendritic spines. Notably, Aβ-os exposure also induces the eEF2 phosphorylation by eEF2 kinase that is known to associate with mGluR5. Aβ-os-induced impairment of LTP is dependent on eEF2 phosphorylation that is increased in brains from both *tg* AD-model mice and AD patient autopsies (Nygaard, 2018).

Altogether these data support the view that mGluR5 activation by specific PAMs facilitates excitotoxic mechanisms causing the death of neurons (Parmentier-Batteur et al., 2014), whereas mGluR5-specific NAMs act neuroprotectively in AD-model animals (Bruno et al., 2017). Administering MPEP, a mGluR5 selective antagonist, prevents this synaptic loss in *tg* AD-model mice (Rammes et al., 2011; Hu et al., 2014; Kumar et al., 2015). In addition, deletion of mGluR5 prevents memory loss in AD-model mice (Hamilton et al., 2014). But to gain these benefits there is a price to pay, which is the negative impact of mGluR5 selective antagonists on activity-dependent synaptic plasticity mechanisms in brain regions that are not affected by AD (Bruno et al., 2017). Of course, the translatability of these interesting results to human AD patients remains a topic worth exploring.

### Group II mGluRs (-2 and -3)

Group II mGluR2 and mGluR3 are mostly localized presynaptically. Depending on the nature of the ligand, mGluR2s signal *via* Gi/o or Gq11 proteins (González-Maeso et al., 2008; Fribourg et al., 2011) and negatively modulate neuronal excitability (Conn and Pin, 1997). Thus, activation of group II mGluRs is endowed with potential neuroprotective properties as it may curtail glutamatergic signaling and mitigate neuronal hyperexcitability (Nicoletti et al., 1996). Stimulation of group II mGluRs inhibits adenylyl cyclase (AC), activates K^+^ channels, and blocks presynaptic voltage-gated calcium channels, thus hindering intracellular Ca²^+^ fluxes and synaptic neurotransmitters release (Benarroch, 2008; Niswender and Conn, 2010). Groups II mGluRs also team with the MAPK and PI3K pathways to confer neuroprotection (D’Onofrio et al., 2001). As mentioned also below, neuroprotection is mediated by transforming growth factor-β1 (TGF-β1) released through astrocytes’ mGluR3 signaling. TGF-β1 binds and activates its membrane receptors coupled with serine/threonine kinase activity thereby inducing the *Smad* signaling cascade. It also synergistically operates with other neurotrophins such as nerve growth factor (NGF), brain-derived neurotrophic factor (BDNF), and glial-derived neurotrophic factor (GDNF) (Caraci et al., 2011b). In the rodents’ thalamus, the selective activation of mGluR2 modulates the inhibition at synapse level of sensory neurons functionally linked to information processing, attention, and cognition (Copeland et al., 2017). Conversely, the selective activation of mGluR2 increases the incidence of neuronal deaths *in vitro* (Corti et al., 2007; Caraci et al., 2011b). Accordingly, a mGluR2-specific NAM hindered the death of ischemia-sensitive neurons in the hippocampal CA1 area, whereas a mGluR2-specific PAM promoted the death both of ischemia-sensitive CA1 neurons and of ischemia-resistant CA3 neurons (Motolese et al., 2015). Recent investigations have revealed the formation of intragroup and intergroup heterodimers between different mGluRs (Doumazane et al., 2011; Rondard et al., 2011; Kammermeier, 2012). New allosteric modulators capable of differentiating homodimers from heterodimers have disclosed the assembly of mGluR2/mGluR4 heterodimers in corticostriatal fibers (Yin et al., 2014).

The mGluR3, whose activation inhibits AC activity and hence cyclic AMP production, is the most abundant astroglial receptor along all the lifetime (Sun et al., 2013). Mounting evidence indicates that mGluR3 upkeeps synaptic homeostasis, including synaptic plasticity and synaptogenesis (see for references: Durand et al., 2013). In addition, activated mGluR3 plays major neuroprotective roles in AD and other neuropathologic conditions. Once added to pure cultures of newborn rat astrocytes, the orthosteric agonists LY379268 or LY354740 specifically activated mGluR3 (rodent astrocytes do not express mGluR2), promoting the production and release of TGF-β and of GDNF (see also above and Caraci et al., 2011a). The same agonists increased the expression of α-secretase, whose activity is essential for APP’s physiological *NAP*. The upshot is an amplified extracellular shedding of the neurotrophic and neuroprotective and GABA_B_R1a agonist sAPP-α (**Figure 1**) (Bruno et al., 1997; Bruno et al., 1998; Corti et al., 2007; Battaglia et al., 2009; Di Liberto et al., 2010; Battaglia et al., 2015; Rice et al., 2019). Moreover, an indirect role for mGluR3 in AD is denoted by the progressive decrease with aging in mGluR2 and mGluR3 expression and, consequently, in their antiamyloidogenic action in hippocampal astrocytes from PDAPP-J20 AD-model mice (Durand et al., 2014). In subsequent studies, the same authors showed that the LY379268-elicited activation of astrocytes’ and neurons’ mGluR3 suppressed or mitigated the Aβ-driven neurotoxicity and death of both neurons and astrocytes. In both cell types, agonist-activated mGluR3 increased the shedding of neuroprotective sAPP-α and the expression of BDNF. In addition, LY379268-activated mGluR3s induced astroglia- and microglia-mediated phagocytosis and removal of Aβs from the extracellular environment. Finally, mGluR3 orthosteric agonists LY379268 or LY404039 suppressed the nitric oxide (NO)-induced death of cultured rat astrocytes *via* the inhibition of AC, which reduced intracellular cAMP levels, the activation of Akt, and the formation of antiapoptotic p65 and c-Rel complexes of the NF-κB family (Durand et al., 2011; Durand et al., 2017).

Conversely, Caraci et al. (2011a) showed that mGluR2 and mGluR3 enhanced neurotoxicity in pure cultures of rat brain neurons challenged with Aβ_1–42_ or with its neurotoxic fragment Aβ_25–35_. However, if the neurons were cocultured with the astrocytes, the activation of mGluR2 and mGluR3 brought about neuroprotective effects through the release of TGF-β_1_ from the astrocytes. TGF-β_1_ is a well-known agent endowed with neuroprotective and anti-inflammatory activities (see also above) in experimental AD-models (Chen et al., 2015) as it also stimulates microglia to scavenge Aβs (Tichauer and von Bernhardi, 2012).

### Group III mGluRs (-4, -6, -7, and -8)

Group III mGluRs (-4, -6, -7, and -8) are mainly localized presynaptically, couple to Gαi/o, and negatively modulate neuronal excitability (Conn and Pin, 1997). They are likely to act as autoreceptors on glutamatergic synaptic terminals and as heteroceptors on GABAergic and other neurotransmitter terminals (Cartmell and Schoepp, 2000; Ferraguti and Shigemoto, 2006). Group III mGluRs stimulation results in AC inhibition, K^+^ channels activation, and block of presynaptic voltage-gated calcium channels, thus decreasing Ca²^+^ flow into cells and neurotransmitters release from synapses (Benarroch, 2008; Niswender et al., 2008). Therefore, their activation elicits potential neuroprotective effects that dampen glutamatergic signaling and inhibit neurotransmitters release thereby mitigating neuronal excitability (Nicoletti et al., 1996). As a particularity, activated mGluR7 stimulates protein kinase C (PKC) or phospholipase C resulting in the inhibition of neuronal calcium channels (Perroy et al., 2000; Pelkey et al., 2007). Brain expression of mGluR4, -7, and -8 is proper of cortical and hippocampal neurons and of synapses located in the basal nuclei (striatum, pallidum), subthalamic nucleus, and substantia nigra (both pars compacta and pars reticularis) (Bruno et al., 1996; Faden et al., 1997; Hovelsø et al., 2012). Instead, mGluR6 expression is exclusive of the retina (Nakajima et al., 1993). The main subcellular location of mGluR7 is at the central area of presynaptic terminals just where the membrane coalesces with synaptic vesicles: this suggests its involvement in the modulation of neurotransmitter release. Conversely, mGluR4 and mGluR8 are placed at the periphery of presynaptic terminals (Shigemoto et al., 1997; Schoepp, 2001; Palucha and Pilc, 2007). Group III mGluRs also cooperate with MAPK and PI3K signaling pathways to impart neuroprotection (Iacovelli et al., 2002). Recently, these receptors have become the focus of therapeutic attempts because they (i) can modulate defective neurotransmission yielding symptomatic improvements through the neuroprotective hindering of multiple neurodegenerative mechanisms and (ii) have more favorable safety and tolerability profiles (Hovelsø et al., 2012). Activation of group III mGluRs by glutamate and/or other agonists is neuroprotective as it inhibits glutamate release from neurons’ presynaptic terminals and from microglia, thus mitigating excitotoxicity; concurrently, astrocytes intensify the uptake of glutamate and microglia increase neurotrophic factors synthesis (see Williams and Dexter, 2014 for an in-depth review on this topic).

Rather few studies exist about the effects on a neurodegenerative disease like AD exerted by group III mGluRs activation *via* broad spectrum agonists and PAMs or inactivation *via* NAMs. Copani et al. (1995) reported that broad-spectrum group III mGluRs agonists L-serine-O-phosphate (L-SOP) and l-2-amino-4-phosphono-butanoate (L-AP4) could lessen the apoptotic death rate of neurons exposed to Aβs. The authors suggested that such agonists would exert neuroprotective effects in AD. Similarly, group III agonist RS-PPG, which activates preferentially mGluR8 and likely also mGluR4, exerted neuroprotective actions on neurons exposed to harmful hypoxic or hypoglycemic conditions (Bruno et al., 2000; Sabelhaus et al., 2000). Notably, acute hypoxia can induce neurons to overproduce lethal amounts of Aβs *via* a mechanism. involving another family C GPCR, the CaSR (Kim et al., 2014; Bai et al., 2015). Besides, PHCCC, a specific mGluR4 PAM, and also a partial antagonist of group I mGluRs, protected cultured cortical mouse neurons against the Aβs-elicited cytotoxicity and NMDAR excitotoxicity (Maj et al., 2003).

But what about the astrocytes? Under basal conditions, rodent (rat and mouse) astrocytes in primary cultures express mGluR4, but neither mGluR7 nor mGluR8 (Phillips et al., 1998; Janssens and Lesage, 2001). However, mGluR8 is expressed by reactive astrocytes adjacent to chronic inflammatory lesions (Geurts et al., 2005). Besong et al. (2002) provided evidence that broad spectrum orthosteric agonists activating group III mGluRs, like L-AP4, 4-phosphonophenylglycine (4-PPG), or L-SOP hindered the expression and secretion of the proinflammatory chemokine RANTES in astrocyte cultures. These beneficial effects of the mGluR4 broad spectrum agonists were counteracted by pretreating the astrocytes cultures with the selective group III mGluRs NAM (R,S)-α-methyl-serine-O-phosphate or with pertussis toxin. Altogether, these findings suggest that such agonists might mitigate neuroinflammation in conditions like AD, multiple sclerosis, and experimental allergic encephalomyelitis.

Extracellular glutamate homeostasis, which is essential for physiological glutamatergic neurotransmission and excitotoxicity prevention, depends on the activity of astrocytes’ transporters like GLT-1 and GLAST (Anderson and Swanson, 2000). Neuroinflammatory conditions associated with a neurodegenerative disease like AD or experimental treatments (e.g. with LPS, MPTP, etc.) reduce astrocytes’ GLT-1- and GLAST-mediated glutamate uptake due to a fall in endogenous antioxidant glutathione (GSH) activity. Broad spectrum group III mGluRs agonists rescue GSH normal levels and restore astrocytes’ GLT-1- and GLAST-mediated glutamate uptake alleviating neuronal excitotoxicity (Yao et al., 2005; Zhou et al., 2006; Foran and Trotti, 2009). Thus, activation of astrocytes’ group III mGluR3 and mGluR5 and also of group II mGluRs by broad spectrum agonists increases GLT-1- and GLAST protein expression and glutamate uptake activity as the signaling of both groups likely involves Gi/o, MAPKs, and PI3K pathways (Aronica et al., 2003; Beller et al., 2011; Williams and Dexter, 2014). The activation of group III mGluRs by wide spectrum agonists also curtails the release of proinflammatory cytokines from activated microglia (Combs et al., 2000). Wide spectrum group III mGluRs agonists also hinder proinflammatory cytokines release, RANTES included, from the astrocytes exposed to neurotoxic agents (Mennicken et al., 1999; Besong et al., 2002), thereby helping mitigate neuroinflammation and reduce neuronal demise. Therefore, one might surmise that the effects of these agonists on astrocytes and microglia would likely impact on the course of AD and perhaps also of other neurodegenerative diseases.

## Calcium Sensing Receptor

The CaSR is a (poly)cationic receptor, as its evolutionary history shows (Riccardi and Kemp, 2012). This is why CaSR’s preferred yet not unique orthosteric agonist is Ca^2+^. A CR region, necessary for receptor activation (Huang et al., 2011; Hendy et al., 2013) connects CaSR’s huge (∼612 amino acids) ECD, the bilobed (LB1 and LB2) VFT to the 7TM domain whose seven transmembrane α-helices (TM1–TM7) are joined by three extracellular and three intracellular loops. Two domains of the CASR’s intracellular C-terminal tail are necessary for CaSR expression at the cell surface and its composite signaling functions *via* G-proteins (see below). The VFT contains the binding pockets for the orthosteric (type I) agonists (Hendy et al., 2013), which besides extracellular Ca^2+^ (Hofer and Brown, 2003), include various divalent and trivalent cations, polyamines, aminoglycoside antibiotics, and cationic polypeptides (Silve et al., 2005; Saidak et al., 2009; Magno et al., 2011; Zhang et al., 2015a). The CaSRs of human cortical astrocytes also specifically bind Aβs, likely at the VFTs (Dal Prà et al., 2014a; Dal Prà et al., 2014b, Dal Prà et al., 2015b). Moreover, X-ray crystallography studies (Geng et al., 2016) have revealed that in the resting state the 3D structure of CaSR’s ECD exhibits an open conformation kept up by PO_4_^3−^ anions. Independently of the presence or absence of Ca^2+^ ions, CaSR activation occurs when an L-α-amino acid closes the VFT groove, triggering the formation of a new homodimer interface between the membrane-proximal LB2 and the CR domains. Ca^2+^ ions stabilize the active state to fully activate the receptor. Indeed, CaSR’s ECD is endowed with four Ca^2+^-binding sites, of which the Ca^2+^ ion at site #4 stabilizes, upon orthosteric agonist binding, the CaSR homodimer’s active conformation (Geng et al., 2016). Importantly, orthosteric agonists also induce the dissociation of inhibitory PO_4_^3−^ anions from the arginine residues acting as their relatively weak binding sites. Thus, the CaSR-inactivating action of bound PO_4_^3−^ anions is overturned (Quinn et al., 1997; Cheng et al., 2004; Geng et al., 2016). As the other GPCRs do, CaSR swings between conformation-varying active and inactive states (Rosenbaum et al., 2011). The changes in conformation due to activation include a rearrangement of the 7TM and ICD domains. The CaSR’s 7TM helical domains can modulate signal transduction. The 7TM’s intracellular loops 2 and 3 are crucially involved in the activation of downstream effectors (Goolam et al., 2014). Besides, various CaSR’s 7TM sites bind allosteric (type II) ligands. The latter include both the aromatic L-α-amino acids and the highly selective *allosteric agonists* or *PAMs*, short-termed *calcimimetics*, and *allosteric antagonists* or *NAMs*, short-termed *calcilytics* (Nemeth, 2002). As will be discussed later, these pharmacological agents offer exciting perspectives in the field of clinical therapeutics. In response to orthosteric ligand binding, the CaSR’s ICD tails interact with G_s_ or G_q/11_ or G_12/13_, or G_i/o_, proteins (Chang et al., 2001; Hofer and Brown, 2003; Conigrave and Ward, 2013), and with β-arrestin 1/2 (Thomsen et al., 2012). Such interactions turn on several signaling pathways (Saidak et al., 2009; Magno et al., 2011), which underlie the receptor’s complex actions and comprise: (i) second messenger-producing enzymes (e.g., AC); (ii) phospholipases A2, C, and D; (iii) protein kinases (e.g. PKCs, MAPKs, AKT); (iv) Ca^2+^ influx *via* TRPC6-encoded receptor-operated channels; and (v) transcription factors (reviewed in Zhang et al., 2015a). Moreover, the intracellular adaptor-related protein complex (AP2) binds the CaSR’s ICD promoting the receptor’s clathrin-mediated endocytosis (Nesbit et al., 2013). Finally, CaSR’s ICD ubiquitylation and phosphorylation modulate the receptor’s recycling, degradation, and desensitization (Zhuang et al., 2012; Breitwieser 2013).

In general, the CaSR preserves systemic Ca^2+^ homeostasis by promptly sensing any changes in the extracellular calcium concentration [Ca^2+^]_e_ and, accordingly, by modulating the amounts of parathyroid hormone (PTH) released from parathyroid glands as well as the reabsorption of Ca^2+^ from kidneys and its deposition in bones (Hofer and Brown, 2003). Dysfunctions of the CaSR severely alter systemic Ca^2+^ homeostasis (Brown, 2007; Hendy et al., 2009). Gain-of-function CaSR mutations result in autosomal dominant hypocalcemia, whereas loss-of-function CaSR mutations cause severe neonatal primary hyperparathyroidism (Hendy et al., 2009; Ward et al., 2012; Hannan et al., 2018).

But, what about the CaSR in the brain? All types of brain neural and cerebrovascular cells express the CaSR, with particular intensely in the hippocampus, an AD-relevant area (Chattopadhyay, 2000; Yano et al., 2004; Noh et al., 2015). Dal Prà et al. (2005) showed that untransformed astrocytes isolated from the adult human temporal cortex and cultured *in vitro* express functional CaSRs, less intensely when proliferating but more strongly when mitotically quiescent. Notably, changes in the growth medium [Ca^2+^]_e_ did not impact on CaSR expression levels by adult human astrocytes. But preservation of systemic Ca^2+^ homeostasis is not the CaSR’s main task in the brain. In fact, fluctuations in [Ca^2+^]_e_ physiologically modulate, *via* corresponding adaptations of CaSR signaling, a variety of neural cells activities like CaSR’s L-amino acid sensing (Conigrave and Hampson, 2006), K^+^ fluxes (Chattopadhyay et al., 1999), proliferation, differentiation, migration of both neurons and oligodendrocytes during growth, and synaptic plasticity and neurotransmission during postnatal life (Bandyopadhyay et al., 2010; Riccardi et al., 2013; Ruat and Traiffort, 2013; Kim et al., 2014; Noh et al., 2015; Tharmalingam et al., 2016).

Remarkably, CNS diseases, such as AD and ischemia/hypoxia/stroke, change the CaSR’s expression levels and hence alter the cellular processes CaSR signaling regulates (Armato et al., 2013; Dal Prà et al., 2014a; Dal Prà et al., 2014b; Bai et al., 2015; Dal Prà et al., 2015b). The first hint that the CaSR might play a role in AD pathogenesis stemmed from the observation that Aβs-elicited peaks of cytosolic [Ca^2+^]_i_ had a killing effect on hippocampal neurons (Brorson et al., 1995). A second clue was the opening of Ca^2+^-permeable nonselective cation channels (NSCCs) by fibrillar Aβ_1–40_ or Aβ_25–35_ in hippocampal neurons of wild type (WT) *CaSR* ^+/+^ rats; notably, this effect could not be replicated in *CaSR* ^−/−^ rats. The authors speculated that Aβs might bind the CaSR because they have, just like polyamines, orderly spaced arrays of positive charges (Ye et al., 1997).

In this regard, the specific formation of plasma membrane Aβs•CaSR complexes and their subsequent endocytosis in cultured cortical untransformed adult human astrocytes could be proven by using the *in situ* proximity ligation assay (isPLA), which reveals the specific formation of stable complexes between two molecules placed within a 30 nm range (Dal Prà et al., 2014a; Dal Prà et al., 2014b; Dal Prà et al., 2015b). The latter results implied that since all types of human neural and cerebrovascular cells express the CaSR, they are vulnerable to the neurotoxic effects driven by pathological Aβ•CaSR signaling (Chiarini et al., 2016). However, it remains to be ascertained whether at the level of CaSR•GABA_B_R1 heterodimers of human cortical astrocytes and neurons Aβs•GABA_B_R1 complexes also form and what their functional roles would be under both physiological and pathological conditions: topics worth investigating further.

Moreover, a genetic analysis study on cohorts of 435 healthy controls and 692 SAD patients showed that an intron 4 polymorphic dinucleotide repeat marker of the *CASR* gene associated with an AD susceptibility, while three nonsynonymous SNPs of exon 7 were linked with an AD propensity only in non-*APOEε4* allele carriers. Hence, variations in the *CASR* gene sequence may impact on SAD susceptibility especially in subjects having no *APOEε4* allele (Conley et al., 2009).

The *CASR* gene P1 and P2 promoters regulate its transcription by binding several transcription factors, including SP1, SP3, STAT1, STAT3, CREB, and NFκB, which concurrently control the expression of other AD-related genes (see for details and references: Chiarini et al., 2016). Therefore, the transcription factors regulation of CaSR expression is tightly linked to the pathophysiology of AD.

It is well known that Aβ_42_-os simultaneously bind to several other CNS cells surface receptors besides the CaSR (see for details and references: Chiarini et al., 2016). Therefore, Aβ_42_-os•CaSR signaling triggers a throng of cellular responses sosme authors include in the so called “*calcium dyshomeostasis*”, such as toxic ROS overrelease from mitochondria, and intracellular Ca^2+^ surges *via* NMDARs’ activation driving further mitochondrial ROS releases (Kam et al., 2014; Jarosz-Griffiths et al., 2016).

However, it must be stressed here that the pathological Aβ_42_-os•CaSR signaling performs much more AD-specific upstream feats than those just mentioned. In fact, it drives the overproduction and overrelease of Aβ_42_-os and p-Tau-os, the two main AD culprits, from human cortical neurons and astrocytes. Moreover, it also induces the production and release of surpluses of other neurotoxic agents, such as NO and VEGF-A, and likely others more, from the adult human cortical astrocytes. Additionally, the pathological Aβ_42_-os•CaSR signaling profoundly suppresses sAPP-α extracellular shedding from human astrocytes and neurons (Chiarini et al., 2016; Chiarini et al., 2017a; Chiarini et al., 2017b). These Aβ-os-elicited noxious effects associate with concurrent upsurges in the expression of APP, BACE1, and CaSR proteins. Remarkably, the crucial upshot of all the mentioned effects of Aβ_42_-os•CaSR signaling is the death of human cortical neurons both *in vitro* (Armato et al., 2013) and in the *in vivo* brain. In the latter, the progressive disconnections of neural circuits—a cause of advancing cognitive decline—and a chronic diffuse reactive neuroinflammation eventually lead to full blown or symptomatic AD (Crimins et al., 2013; Kayed and Lasagna-Reeves, 2013; Medeiros et al., 2013).

Moreover, a study using *3xTg* AD-model mice showed that the amount of brain CaSR immunoreactivity progressively increased with age, particularly in areas where Aβ_42_ fibrils accumulate most, such as the hippocampi. Thus, local fibrillar Aβ_42_ buildup and CaSR expression raise in parallel in both Aβ-exposed human cortical neurons and astrocytes cultured *in vitro* and in the hippocampi of *3xTg* AD-model mice (Armato et al., 2013; Chiarini et al., 2016; Gardenal et al., 2017). This soaring expression of neural cells’ CaSRs associates with a declining expression of inhibitory GABA_B_R1as (Chang et al., 2007; Kim et al., 2014).

Whereas GABA_B_ and taste receptors obligatorily function as heterodimers (Jones et al., 1998; Nelson et al. 2002), mGluRs and CaSR function both as disulfide-linked homodimers (Zhang et al., 2001; Pidasheva et al., 2006) and as CaSR/GABA_B_Rs, CaSR/mGlu1αR and CaSR/mGlu5R heterodimers (Gama et al., 2001). Ectopic overexpression and coimmunoprecipitation studies revealed that CaSR/GABA_B_R1a heterodimers do affect CaSR protein expression in opposing ways. The total and cell surface expression and signaling of the CaSRs were suppressed by coexpressing GABA_B_R1as, being instead increased (i) by co-expressing GABA_B_2Rs; (ii) by knocking out GABA_B_R1a in mouse brains; and (iii) by deleting GABA_B_R1a in cultured hippocampal neurons. The GABA_B_Rs and CaSRs form heterodimers as soon as they are synthesized, since these protein complexes are already detectable around the cells’ nuclei and in the endoplasmic reticulum. In such early complexes GABA_B_Rs bind an immature form of the CaSR. Clearly, GABA_B_R1a and GABA_B_R2 subunits compete for the CASRs. The CaSR/GABA_B_R heterodimers appear to have altered pharmacological properties with respect to the prevailing CaSR homodimers. Results gained using (i) the GABA_B_Rs agonists baclofen and GABA, (ii) the GABA_B_R1a antagonist CGP-3548, and (iii) GABA_B_R1a expression knockdown in cultured mouse growth plate chondrocytes indicated that GABA_B_R1a can elicit both CaSR-independent and CaSR-mediated actions. However, divergent results gained from different experimental models suggested that an endogenous expression or a targeted overexpression of one or more of these receptors, coexisting differences in ligands and in their relative quantities and in downstream intracellular signaling pathways could elicit unlike upshots under various physiological and/or pathological conditions (Gama et al., 2001; Chang et al., 2007). During the initial phases of disease progression in AD-model animals, the decline of GABA_B_R1s’ availability, which concurs with CaSR’s overexpression, induced a *neuronal hyperactivity* in hippocampal and cerebrocortical circuits, whose upshot was functional impairment (Busche and Konnerth 2015). The mechanism(s) underlying this loss of neuronal working capability remain(s) unclear: an overconsumption of O_2_ on the part of the hyperactive neurons might be a contributory factor. Nothing is so far known about the existence and pathophysiological roles of CaSR heterodimers in cortical human untransformed astrocytes and neurons. Therefore, to-date the impacts (if any) the CaSR/GABA_B_Rs and CaSR/mGluRs heterodimers might exert on human AD’s course and on anti-AD therapeutic approaches remain to be assessed.

**Figure 3:**
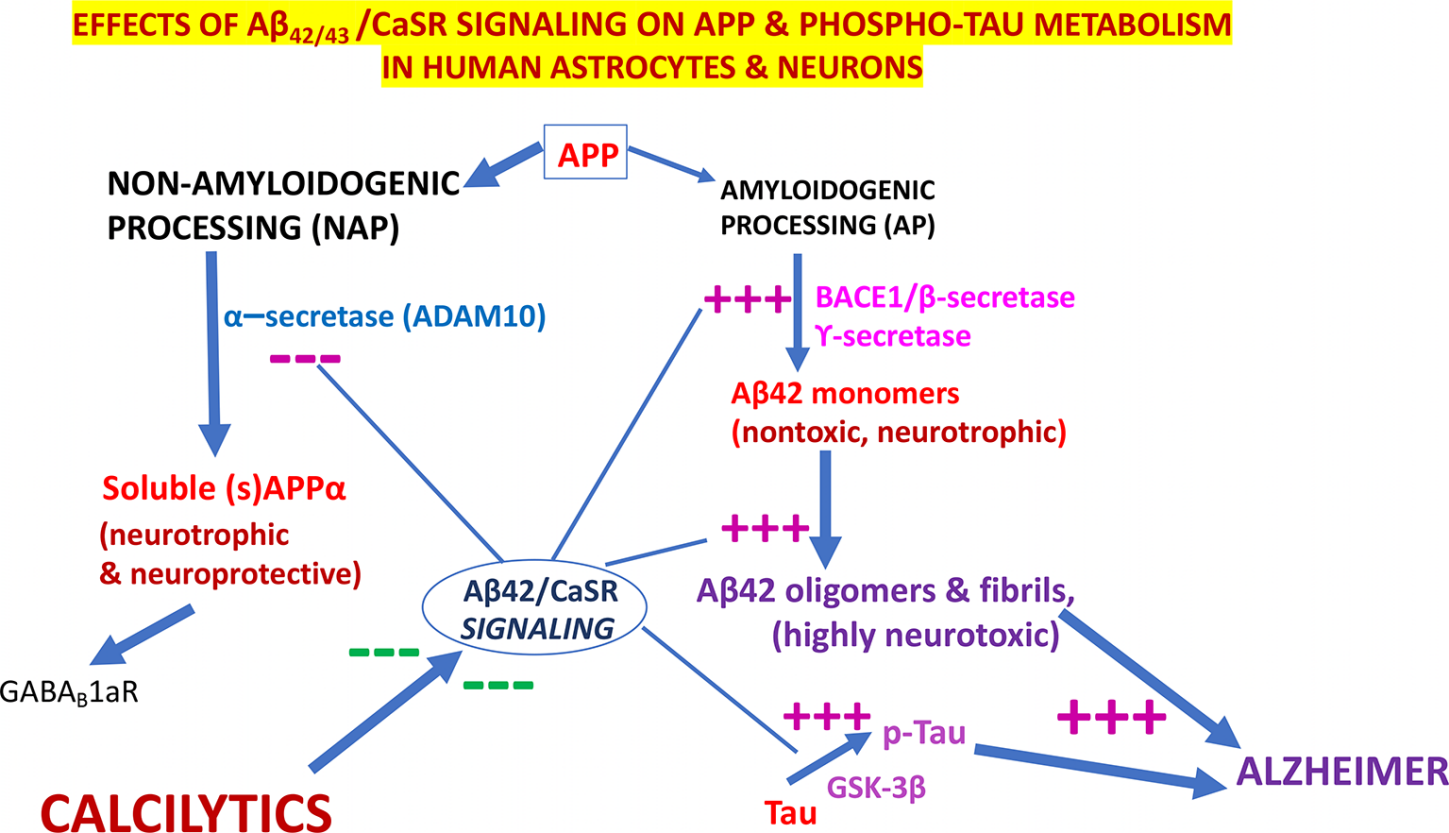
The pathological effects of Aβ•CaSR signaling on the metabolic processing of amyloid precursor protein (APP) and Tau proteins in untransformed cortical human astrocytes and neurons and their complete suppression by highly selective calcium-sensing receptor (CaSR) negative allosteric modulators (NAMs) (or calcilytics). Under physiological conditions, the *NAP* of APP largely prevails in cortical human astrocytes and neurons. Conversely, the pathological Aβ•CaSR signaling hugely enhances the APP holoprotein’s *AP* at the expense of *NAP* in both human cell types. This leads to a surplus synthesis, intracellular accumulation, and extracellular release of Aβ_42_-os. The latter spread extracellularly to bind and activate the signaling of the CaSRs of adjoining teams of astrocytes and neurons (Chiarini et al., 2017a). Such self-sustaining vicious cycles amplify and propagate the pathological Aβ•CaSR signaling and its neurotoxic effects to wider and wider cortical areas. The Aβ•CaSR signaling also increases the activity of the glycogen synthase kinase-3β (GSK-3β), which strongly phosphorylates Tau proteins at amino acid sites typical of Alzheimer’s disease (AD). The thus hyperphosphorylated Tau proteins also form oligomers (p-Tau-os) that are next released extracellularly within exosomes (not shown), thereby starting the tauopathy typical of AD. Other noxious effects of Aβ•CaSR signaling, such as increases in the synthesis and release of nitric oxide (NO) and vascular endothelial growth factor-A (VEGF-A), and other proinflammatory agents are not shown here for the sake of clarity. The crucial upshot of the harming effects of pathological Aβ•CaSR signaling is the progressive death of the cortical human neurons crucially involved in memories and cognition processing. In a most striking fashion, highly selective CaSR NAMs (calcilytics) suppress *all* the just mentioned neurotoxic effects brought about by pathological Aβ•CaSR signaling thus restoring the APP’s *NAP*, Tau, NO, and VEGF-A to their physiological settings and consequently preserving the viability and function of human neurons notwithstanding the presence of Aβ peptides. Hence, NAMs could stop AD progression, safeguard the survival of the cortical human neurons, and preserve the memories, cognitive and coping capabilities of the patients. — blocking effects; +++, stimulating effects.

Notably, in cortical adult human astrocytes the pathological Aβ•CaSR signaling heavily affects the APP holoprotein metabolism significantly deflecting it from its physiological *NAP* (**Figure 3**). APP’s *NAP* typically obstructs the *de novo* production of Aβ_42_s/Aβ_42_-os since the α-secretases (mainly ADAM 10) cut the APP molecule just within the Aβ_42_ amino acid sequence (Kuhn et al., 2010) (**Figures 1** and **3**). Notably, APP’s *NAP* prevails over APP’s *AP* in untreated (control) cortical adult human astrocytes, which directly shed all the sAPP-α they produce into the environment while secreting only tiny amounts of monomeric Aβ_42_ (Chiarini et al., 2017b). Hence, it has been posited that by constitutively releasing substantial amounts of sAPP-α, which is an agonist of GABA_B_R1as (Rice et al., 2019), human astrocytes could continually abate any noxious neuronal hyperexcitability. Under the same basal conditions, CaSR signaling is only modulated by extracellular cations levels, particularly by the [Ca^2+^]_e_. On the other hand, a dramatic change in this mechanism occurs when increased quantities of exogenous Aβs bind the human astrocytes’ (and neurons’) CaSRs and activate their pathological signaling that strongly promotes APP’s *AP* over APP’s *NAP* (**Figure 3**). This leads to an excess production, accumulation, and secretion of neurotoxic Aβ_42_/Aβ_42_-os from the cortical astrocytes and from the neurons in which an alike APP’s *AP* mechanism operates (Armato et al., 2013). Concurrently, the astrocytes’ and neurons’ intense extracellular shedding of sAPP-α is curtailed by ∼70%, while sAPP-α abnormally accumulates within the cells (Chiarini et al., 2017a). On the basis of such results the authors posited that an ongoing Aβ•CaSR signaling that would spread in vicious cycles from teams to teams of “*master*” astrocytes’ and “*client*” neurons could cause a substantial loss of the neurotrophic and neuroprotective effects otherwise brought about by extracellularly shed sAPP-α, including its agonistic action on GABA_B_R1as, thereby favoring a harmful neuronal hyperexcitability. In addition, the Aβ_42_/Aβ_42_-os-exposed human astrocytes and neurons could simultaneously release increasing amounts of neurotoxic Aβ_42_-os (Armato et al., 2013), p-Tau-os (within exosomes) (Chiarini et al., 2017a), NO, VEGF-A (Dal Prà et al., 2005; Chiarini et al., 2010; Dal Prà et al., 2014a; Dal Prà et al., 2014b; Chiarini et al., 2016). and likely other noxious agents. Therefore, it would not be surprising that under such dire circumstances cortical human neurons keep losing synapses and consequently die. Interestingly, in line with the just mentioned findings, CSF levels of sAPP-α significantly decrease in LOAD/SAD patients (Lewczuk et al., 2010, which indirectly confirms the substantial fall of its extracellular shedding from human astrocytes (Chiarini et al., 2017b).

### CaSR NAMs as Potential Anti-AD Therapeutics

As mentioned above, several PAMs and NAMs of the CaSR are available. L-α-amino acids with an aromatic ring and positively charged amino groups (NH^3+^) are naturally occurring CaSR PAMs (Lee et al., 2007). Synthetic phenylalkylamine CaSR PAMs (“*calcimimetics*”; e.g. AMG 416, Cinacalcet, and NPS R-568) having two-to-four aromatic rings and NH^3+^ groups have been synthesized. PAMs augment the CaSR’s sensitivity to activation by [Ca^2+^]_e_ and hence lower the EC_50_ for [Ca^2+^]_e_. Notably, three CaSR PAMs, i.e. Evocalcet, Etelcalcitide, and Cinacalcet, have successfully reached the clinical use to mitigate primary and secondary hyperparathyroidism and tumor-elicited hypercalcemias (Nemeth and Goodman, 2016).

CaSR NAMs are small amino-alcohol molecules like NPS 2143 (Nemeth et al., 2001), Calhex 231 (Kessler et al., 2006), NPSP795 (Gafni et al., 2015), or quinazolinones like ATF936 and AXT914 (Wildler et al., 2010). CaSR NAMs right-shift the [Ca^2+^]_e_ response curve decreasing the CaSR sensitivity to [Ca^2+^]_e_ and thus increasing the EC_50_ for [Ca^2+^]_e_ (Ferry et al., 1997; Huang and Breitwieser, 2007). As previously anticipated, both PAMs and NAMs bind the 7TM domain of the CaSR. The CaSR binding pockets of NAMs and PAMs partially overlap but are not identical. NAMs bind between the TM3 and TM5 loops, whereas both PAMs and NAMs attach between the TM6 and TM7 loops (Petrel et al., 2003; Petrel et al., 2004; Miedlich et al., 2004). It has been shown that point mutated residues of the 7TM helices (i.e. Phe^668^, Phe^684^, Trp^818^, Phe^821^, Glu^837^, and Ile^841^) lessen the antagonism of CaSR NAM NPS 2143 (Petrel et al., 2003; Petrel et al., 2004; Miedlich et al., 2004). CaSR’s allosteric agonism and antagonism are modulated *via* the involvement of distinct amino acids and mechanisms (see for further details Leach et al., 2016; Keller et al., 2018). The identification of synthetic allosteric modulators of the CaSR has prompted searches for their therapeutic applications in diseases in which the CaSR signaling is dysfunctional (Hannan et al., 2018). However, hitherto the therapeutic potentials of both PAMs and NAMs of the CaSR have been only modestly exploited (Nemeth, 2013; Saidak et al., 2009; Widler, 2011; Ward et al., 2012). Like other GPCRs, CaSRs exhibit the “*ligand-biased signaling*” feature, i.e. in a certain type of cell a signaling pathway may be steadily picked up over the others according to the specific ligand involved (Leach et al., 2015). Interestingly, NAMs and PAMs too can induce this biased signaling, which in future might allow to therapeutically target a speciﬁc cell type over others (see for details: Davey et al., 2012; Leach et al., 2015; Hannan et al., 2018).

Let’s zero in on an important NAMs’ feature: they enhance parathyroid hormone (PTH) secretion from the parathyroid glands and increase blood calcium levels (calcemia) (Nemeth, 2004; Nemeth, 2013). Several phase II clinical trials were undertaken to assess CaSR NAMs potential therapeutic efficacy in women with postmenopausal osteoporosis based on the assumption that the released PTH would stimulate osteogenic processes. However, these trials failed because NAMs induced a several hour-lasting oversecretion of PTH that stimulated both osteogenic and osteolytic processes in the osteoporotic bones. These failures prompted to search for new CaSR NAMs inducing a lesser and shorter-lasting PTH release (Nemeth, 2013; Riccardi and Kemp, 2012; Davey et al., 2012; Ward et al., 2012). The same failures also demoted CaSR NAMs from the drugs potentially beneficial in humans even because of the modest hypercalcemia (hyperparathyroidism) they induced. However, attempts were performed to treat hypoparathyroidism and autosomal dominant hypocalcemia (ADH) driven by gain-of-function CaSR mutations with CaSR NAMs (Nemeth, 2013; White et al., 2009; Letz et al., 2010; Park et al., 2013; Nemeth and Goodman, 2016). The use of CaSR NAMs was also considered in cases of breast and prostate carcinomas to prevent bone metastases, which are established through a CaSR-mediated signaling (Liao et al., 2006; Mihai et al., 2006). Other potential therapeutic uses of CaSR NAMs have included asthma attacks (Yarova et al., 2015); pulmonary artery idiopathic hypertension (Yamamura et al., 2012; Yamamura et al., 2015); stroke (Kim et al., 2014); and, last but not least, LOAD/SAD and EOFAD (Armato et al., 2013; Chiarini et al., 2016; Chiarini et al., 2017a; Chiarini et al., 2017b; Chiarini et al., 2017c).

The use of CaSR NAMs as therapeutics in SAD/LOAD and EOFAD is supported by the results gained from preclinical AD models “in Petri dishes” made up by untransformed human cortical astrocytes and/or neurons. In fact, a 30-min administration of a CaSR NAM, be it NPS 2143 or NPS 89696, completely suppressed all the above-mentioned neurotoxic responses evoked by the pathological Aβ•CaSR signaling (Chiarini et al., 2010; Armato et al., 2013; Dal Prà et al., 2014b; Dal Prà et al., 2015a; Chiarini et al., 2016; Chiarini et al., 2017c). Therefore, the authors posited that *in vivo* administered CaSR NAMs would (A) preserve the shedding of neurotrophic and neuroprotective and GABA_B_R1a agonist sAPP-α from the plasma membranes of astrocytes and likely neurons, thereby (i) obstructing the amyloidogenesis from APP and hence the cerebral accumulation of neurotoxic soluble Aβ_42_-os and fibrillar Aβ_42_ polymers, and (ii) abating the noxious neuronal hyperexcitability *via* sAPP-α•GABA_B_R1a signaling; (B) suppress the surplus synthesis and exosomal intrabrain dissemination of neurotoxic p-Tau-os and the consequent hypertoxic effects elicited by combined actions of the Aβ_42_-os/p-Tau-os duet (Ittner and Götz, 2011; Chiarini et al., 2017a); (C) reduce the increased synthesis and secretion of neurotoxic amounts of NO, VEGF-A, and likely other neurotoxic agents; (D) suppress any other harmful effects elicited by Aβ•CaSR signaling in oligodendrocytes, microglia, cerebrovascular cells of any kind; and (E) safeguard the blood–brain barrier (BBB) functional integrity. The above *in vitro* results also indicate that NAM efficacy persists notwithstanding a continued presence of soluble Aβ-os, fibrillar Aβs, and p-Tau-os (Chiarini et al., 2017a; Chiarini et al., 2017b; Chiarini et al., 2017c). Therefore, it is likely that CaSR NAMS could safeguard *in vivo*, as they do *in vitro* (Armato et al., 2013), the viability and functions of the cortical human neurons preserving the integrity of critical cognition-essential upper cerebral cortical regions (Choi et al., 2013; Lee et al., 2013; Barateiro et al., 2016). In brief, CaSR NAMs would uphold the patients’ ability to record and recover memories and to deal with their daily needs. Most important, the relatively cheap to synthesize CaSR NAMs appears to be the so far unique class of anti-AD therapeutics capable of concurrently targeting the multiple noxious effects triggered by pathological Aβs•CaSR signaling in human neurons, astrocytes, and the other brain cell types (Chiarini et al., 2010; Dal Prà et al., 2011; Armato et al., 2013; Dal Prà et al., 2014b; Dal Prà et al., 2015a; Chiarini et al., 2016).

Ischemic neuronal injury is known to locally generate Aβs surpluses (Ishimaru et al., 1996). More recent studies showed that the intraventricular administration of CaSR NAMs did decrease the death of neurons in the cortical *penumbra* zone of animal models of ischemia/hypoxia/stroke by effectively suppressing the concurrent *acute* increase in the local Aβ-os production (Kim et al., 2014; Bai et al., 2015). These results further strengthen the idea that pathological Aβ•CaSR signaling is crucially involved in both acute (ischemia/stroke) and chronic (AD) conditions causing neuronal death (Armato et al., 2013; Dal Prà et al., 2015a; Chiarini et al., 2016).

*Fyn* kinase inhibitor Saracatinib (AZD0530) and NMDAR inhibitors Memantine and Nitromemantine were endorsed as drugs to counteract the neurotoxicity driven by the extracellular accumulation of Aβ_42_-os (Kaufman et al., 2015). However, CaSR NAMs act on Aβ•CaSR signaling well upstream of *Fyn* and NMDARs. In addition, CaSR NAMs obstruct any cytotoxic effects and likely also any impediments to proliferation and differentiation of neural stem cells in the dentate gyrus subgranular zone (Unger et al., 2016).

Concerning their most salient pharmacological characteristics, because of their lipid-soluble chemical structures and limited numbers of electrical charges, CaSR NAMs traverse the BBB. They can be administered by any route: hitherto the oral route has been the preferred one for clinical trials. Rodents could endure NAM NPS 2143 administration with no serious off-target effects being reported (Nemeth, 2002; Kim et al., 2014). During the failed phase I and phase II clinical trials assessing NAMs antiosteoporosis activity, human subjects also satisfactorily tolerated the administration of novel NPS 2143 derivatives, which affected PTH release less intensely (no record was taken concerning any brain-related effects). In general, the safety data collected from the clinical trials of CaSR NAMs did not record any major side-effect. Obviously, the calcemia levels had to be checked periodically due to NAM-elicited increases in plasma PTH levels (Nemeth and Shoback, 2013).

## Conclusions

This survey--necessarily short given the huge amount of literature concerning this verily fascinating topic--leads us to several closing considerations. First and foremost, a lot of data from AD-model animals had to be forcibly mentioned because analogous human data are not available. Therefore, there is still quite a lot to discover and learn about the physiological roles of family C GPRS particularly in relation to human CNS and other viscera. Second, it is undeniable that some of these GPCRs could play central roles on human AD. Our work has been mainly, but not exclusively, based upon the experimental exploitation of human cortical astrocyte cultures and has focused on the pivotal role pathological Aβ•CaSR signaling exerts on the onset and progression of AD and on the potentially beneficial therapeutic effects CaSR NAMs could exert in LOAD/SAD patients. The interactions of CaSR heterodimers with other family C GPCRs, e.g. GABA_B_Rs and group I mGluRs, still constitute a mostly unexplored field of endeavor and their impact on AD onset and progression (if any) needs to be clarified. Notably, even in the gene mutations-driven EOFAD, CaSR NAMs could bring to bear mitigating and life-lengthening upshots by suppressing the additional aggravating consequences brought about by the concurrent Aβ•CaSR signaling adding up to those stemming from the mutated genes. Third, any possible AD-promoting effects of CaSR PAMs (calcimimetics) in humans should be thoroughly investigated since in our preclinical *in vitro* AD model PAM NPS R-568 significantly increased Aβ_42_/Aβ_42_-os release from untransformed human adult cortical astrocytes (Armato et al., 2013). Fourth, we wish to add a last comment about CaSR NAMs as candidate therapeutics for human AD. For reasons pertaining to normal physiology, CaSR NAMs failed their initial task as antiosteoporosis therapeutics (Nemeth, 2004; Nemeth, 2013; Nemeth and Shoback, 2013; Nemeth and Goodman, 2016). Moreover, the induction of a mild hypercalcemia by CaSR NAMs has been a bit too much stressed as “hyperparathyroidism” creating a prejudice against their use in humans. Nevertheless, one should remember that no drug is devoid of unwanted and/or off-target effects: the chemotherapeutics administered to oncological patients are a striking example of this. Therefore, CaSR NAMs’ rather slight off-target effects, chiefly the mild controllable hypercalcemia, should be objectively weighed against CaSR NAMs’ crucial capability of averting the worsening loss of memories and cognitive abilities, including recognition of the self, and the later unavoidable demise AD would inexorably deliver.

## Author Contributions

All the authors i.e. IDP, UA, and AC, equally contributed to the bibliographic searches and to the conception, discussion, and writing of the manuscript.

## Funding

This work was supported by the FUR (Fondo Universitario per la Ricerca) 2018 of the Ministry of Italian University and Research to IDP and AC.

## Conflict of Interest

The authors declare that the research was conducted in the absence of any commercial or financial relationships that could be construed as a potential conflict of interest.

## References

[B1] Alzheimer’s Association Report (2018). 2018 Alzheimer’s disease facts and figures. Alzheimers Dement. 14 (3), 367–429. 10.1016/j.jalz.2018.02.001

[B2] Alagarsamy S. Marino M. J. Rouse S. T. Gereau R. W. Heinemann S. F. Conn P. J. (1999). Activation of NMDA receptors reverses desensitization of mGluR5 in native and recombinant systems. Nat. Neurosci. 2 (3), 234–240. 10.1038/6338 10195215

[B3] Alexander S. P. Christopoulos A. Davenport A. P. Kelly E. Marrion N. V. Peters J. A. (2017). The concise guide to pharmacology 2017/18: G Protein-c coupled Receptors. Br. J. Pharmacol. 174 Suppl 1, S17–S129. 10.1111/bph.13878 29055040 PMC5650667

[B4] Ameen-Ali K. E. Wharton S. B. Simpson J. E. Heath P. R. Sharp P. Berwick J. (2017). Review: neuropathology and behavioural features of transgenic murine models of Alzheimer’s disease. Neuropathol. Appl. Neurobiol. 43 (7), 553–570. 10.1111/nan.12440 28880417

[B5] Anderson C. M. Swanson R. A. (2000). Astrocyte glutamate transport: review of properties, regulation, and physiological functions. Glia 32, 1–14. 10.1002/1098-1136(200010)32:1<1::AID-GLIA10>3.0.CO;2-W 10975906

[B6] Aniksztejn L. Sciancalepore M. Ben Ari Y. Cherubini E. (1995). Persistent current oscillations produced by activation of metabotropic glutamate receptors in immature rat CA3 hippocampal neurons. J. Neurophysiol. 73 (4), 1422–1429. 10.1152/jn.1995.73.4.1422 7643157

[B7] Armato U. Chiarini A. Chakravarthy B. Chioffi F. Pacchiana R. Colarusso E. (2013). Calcium-sensing receptor antagonist (calcilytic) NPS 2143 specifically blocks the increased secretion of endogenous Aβ_42_ prompted by exogenous fibrillary or soluble Aβ_25-35_ in human cortical astrocytes and neurons-therapeutic relevance to Alzheimer’s disease. Biochim. Biophys. Acta 1832 (10), 1634–1652. 10.1016/j.bbadis.2013.04.020 23628734

[B8] Aronica E. Gorter J. A. Ijlst-Keizers H. Rozemuller A. J. Yankaya B. Leenstra S. (2003). Expression and functional role of mGluR3 and mGluR5 in human astrocytes and glioma cells: opposite regulation of glutamate transporter proteins. Eur. J. Neurosci. 17, 2106–2118. 10.1046/j.1460-9568.2003.02657.x 12786977

[B9] Attucci S. Carlà V. Mannaioni G. Moroni F. (2001). Activation of type 5 metabotropic glutamate receptors enhances NMDA responses in mice cortical wedges. Br. J. Pharmacol. 132, 799–806. 10.1038/sj.bjp.0703904 11181420 PMC1572635

[B10] Austin P. J. Betts M. J. Broadstock M. O’Neill M. J. Mitchell S. N. Duty S. (2010). Symptomatic and neuroprotective effects following activation of nigral group III metabotropic glutamate receptors in rodent models of Parkinson’s disease. Br. J. Pharmacol. 160, 1741–1753. 10.1111/j.1476-5381.2010.00820.x 20649576 PMC2936845

[B11] Awad H. Hubert G. W. Smith Y. Levey A. I. Conn P. J. (2000). Activation of metabotropic glutamate receptor 5 has direct excitatory effects and potentiates NMDA receptor currents in neurons of the subthalamic nucleus. J. Neurosci. 20 (21), 7871–7879. 10.1523/JNEUROSCI.20-21-07871.2000 11050106 PMC6772731

[B12] Babaev O. Piletti Chatain C. Krueger-Burg D. (2018). Inhibition in the amygdala anxiety circuitry. Exp. Mol. Med. 50 (4), 18. 10.1038/s12276-018-0063-8 29628509 PMC5938054

[B13] Bai S. Mao M. Tian L. Yu Y. Zeng J. Ouyang K. (2015). Calcium-sensing receptor mediated the excessive generation of β-amyloid peptide induced by hypoxia *in vivo* and *in vitro* . Biochem. Biophys. Res. Commun. 459, 568–573. 10.1016/j.bbrc.2015.02 25747709

[B14] Ballester-Rosado C. J. Albright M. J. Wu C. S. Liao C. C. Zhu J. Xu J. (2010). mGluR5 in cortical excitatory neurons exerts both cell-autonomous and –non autonomous influences on cortical somatosensory circuit formation. J. Neurosci. 30 (50), 16896–16909. 10.1523/JNEUROSCI.2462-10.2010 21159961 PMC3008407

[B15] Bandyopadhyay S. Tfelt-Hansen J. Chattopadhyay N. (2010). Diverse roles of extracellular calcium-sensing receptor in the central nervous system. J. Neurosci. Res. 188 (10), 2073–2082. 10.1002/jnr.22391 20336672

[B16] Barateiro A. Brites D. Fernandes A. (2016). Oligodendrocyte development and myelination in neurodevelopment: molecular mechanisms in health and disease. Curr. Pharm. Des. 22, 656–679. 10.2174/1381612822666151204000636 26635271

[B17] Battaglia G. Molinaro G. Riozzi B. Storto M. Busceti C. L. Spinsanti P. (2009). Activation of mGlu3 receptors stimulates the production of GDNF in striatal neurons. PLoS One 4 (8), e6591. 10.1371/journal.pone.0006591 19672295 PMC2719807

[B18] Battaglia G. Riozzi B. Bucci D. Di Menna L. Molinaro G. Pallottino S. (2015). Activation of mGlu3 metabotropic glutamate receptors enhances GDNF and GLT-1 formation in the spinal cord and rescues motor neurons in the SOD-1 mouse model of amyotrophic lateral sclerosis. Neurobiol. Dis. 74, 126–136. 10.1016/j.nbd.2014.11.012 25434487

[B19] Beller J. A. Gurkoff G. G. Berman R. F. Lyeth B. G. (2011). Pharmacological enhancement of glutamate transport reduces excitotoxicity *in vitro* . Restor. Neurol. Neurosci. 29, 331–346. 10.3233/RNN-2011-603 21846950

[B20] Benarroch E. E. (2008). Metabotropic glutamate receptors. Neurology 70, 964–968. 10.1212/01.wnl.0000306315.03021.2a 18347319

[B21] Benes F. M. Berretta S. (2001). GABAergic interneurons: implications for understanding schizophrenia and bipolar disorder. Neuropsychopharmacology 25 (1), 1–27. 10.1016/S0893-133X(01)00225-1 11377916

[B22] Bennouar K.-E. Uberti M. A. Melon C. Bacolod M. D. Jimenez H. N. Cajina M. (2013). Synergy between L-DOPA and a novel positive allosteric modulator of metabotropic glutamate receptor 4: implications for Parkinson’s disease treatment and dyskinesia. Neuropharmacology 66, 158–169. 10.1016/j.neuropharm.2012.03.022 22491024

[B23] Berger J. V. Dumont A. O. Focant M. C. Vergouts M. Sternotte A. Calas A. G. (2012). Opposite regulation of metabotropic glutamate receptor 3 and metabotropic glutamate receptor 5 by inflammatory stimuli in cultured microglia and astrocytes. Neuroscience 205, 29–38. 10.1016/j.neuroscience.2011.12.044 22245498

[B24] Besong G. Battaglia G. D’Onofrio M. Di Marco R. Ngomba R. T. Storto M. (2002). Activation of group III metabotropic glutamate receptors inhibits the production of RANTES in glial cell cultures. J. Neurosci. 22, 5403–5411. 20026585 12097492 10.1523/JNEUROSCI.22-13-05403.2002 PMC6758236

[B25] Bettler B. Kaupmann K. Mosbacher J. Gassmann M. (2004). Molecular structure and physiological functions of GABA(B) receptors. Physiol. Rev. 84 (3), 835–867. 10.1152/physrev.00036.2003 15269338

[B26] Black J. W. Leff P. (1983). Operational models of pharmacological agonism. Proc. R. Soc. Lond. B Biol. Sci. 220 (1219), 141–162. 10.1098/rspb.1983.0093 6141562

[B27] Bloom G. S. (2014). Amyloid-β and tau: the trigger and bullet in Alzheimer disease pathogenesis. JAMA Neurol. 71 (4), 505–508. 10.1001/jamaneurol.2013.5847 24493463 PMC12908160

[B28] Borgkvist A. Avegno E. M. Wong M. Y. Kheirbek M. A. Sonders M. S. Hen R. (2015). Loss of striatonigral GABAergic presynaptic inhibition enables motor sensitization in parkinsonian mice. Neuron 87 (5), 976–988. 10.1016/j.neuron.2015.08.022 26335644 PMC4559856

[B29] Bour A. Little S. Dodart J.-C. Kelche C. Mathis C. (2004). A secreted form of the β-amyloid precursor protein (sAPP 695) improves spatial recognition memory in OF1 mice. Neurobiol. Learn. Mem. 81, 27–38. 10.1016/S1074-7427(03)00071-6 14670356

[B30] Bräuner-Osborne H. Wellendorph P. Jensen A. A. (2007). Structure, pharmacology and therapeutic prospects of family C G-protein coupled receptors. Curr. Drug. Targets 8 (1), 169–184. 10.2174/138945007779315614 17266540

[B31] Breitwieser G. E. (2013). The calcium sensing receptor life cycle: trafficking, cell surface expression, and degradation. Best Pract. Res. Clin. Endocrinol. Metab. 27 (3), 303–313. 10.1016/j.beem.2013.03.003 23856261

[B32] Brorson J. R. Bindokas V. P. Iwama T. Marcuccilli C. J. Chisholm J. C. Miller R. J. (1995). The Ca2+ influx induced by beta-amyloid peptide 25-35 in cultured hippocampal neurons results from network excitation. J. Neurobiol. 26 (3), 325–338. 10.1002/neu.480260305 7775966

[B33] Brown E. M. (2007). Clinical lessons from the calcium-sensing receptor. Nat. Clin. Pract. Endocrinol. Metab. 3, 122–133. 10.1038/ncpendmet0388 17237839

[B34] Bruno V. Copani A. Bonanno L. Knoepfel T. Kuhn R. Roberts P. J. (1996). Activation of group III metabotropic glutamate receptors is neuroprotective in cortical cultures. Eur. J. Pharmacol. 310, 61–66. 10.1016/0014-2999(96)00358-5 8880068

[B35] Bruno V. Sureda F. X. Storto M. Casabona G. Caruso A. Knopfel T. (1997). The neuroprotective activity of group-II metabotropic glutamate receptors requires new protein synthesis and involves a glial-neuronal signaling. J. Neurosci. 17 (6), 1891–1897. 10.1523/JNEUROSCI.17-06-01891.1997 9045718 PMC6793767

[B36] Bruno V. Battaglia G. Casabona G. Copani A. Caciagli F. Nicoletti F. (1998). Neuroprotection by glial metabotropic glutamate receptors is mediated by transforming growth factor-beta. J. Neurosci. 18 (23), 9594–9600. 10.1523/JNEUROSCI.18-23-09594.1998 9822720 PMC6793276

[B37] Bruno V. Battaglia G. Ksiazek I. van der Putten H. Catania M. V. Giuffrida R. (2000). Selective activation of mGlu4 metabotropic glutamate receptors is protective against excitotoxic neuronal death. J. Neurosci. 20, 6413–6420. 10.1523/JNEUROSCI.20-17-06413.2000 10964947 PMC6772963

[B38] Bruno V. Battaglia G. Copani A. D’Onofrio M. Di Iorio P. De Blasi A. (2001). Metabotropic glutamate receptor subtypes as targets for neuroprotective drugs. J. Cereb. Blood Flow Metab. 21 (9), 1013–1033. 10.1097/00004647-200109000-00001 11524608

[B39] Bruno V. Caraci F. Copani A. Matrisciano F. Nicoletti F. Battaglia G. (2017). The impact of metabotropic glutamate receptors into active neurodegenerative processes: a “dark side” in the development of new symptomatic treatments for neurologic and psychiatric disorders. Neuropharmacology 115, 180–192. 10.1016/j.neuropharm.2016.04.044 27140693

[B40] Busche M. A. Chen X. Henning H. A. Reichwald J. Staufenbiel M. Sakmann B. (2012). Critical role of soluble amyloid-β for early hippocampal hyperactivity in a mouse model of Alzheimer’s disease. Proc. Natl. Acad. Sci. U S A. 109 (22), 8740–8745. 10.1073/pnas.1206171109 22592800 PMC3365221

[B41] Busche M. A. Grienberger C. Keskin A. D. Song B. Neumann U. Staufenbiel M. (2015). Decreased amyloid-β and increased neuronal hyperactivity by immunotherapy in Alzheimer’s models. Nat. Neurosci. 18 (12), 1725–1727. 10.1038/nn.4163 26551546

[B42] Busche M. A. Konnerth A. (2015). Neuronal key defect in Alzheimer’s disease? Bioessays 37, 624–632. 10.1002/bies.201500004 25773221

[B43] Butterfield D. A. Boyd-Kimball D. (2018). Redox proteomics and amyloid β-peptide: insights into Alzheimer disease. J. Neurochem. 10.1111/jnc.14589 PMC6417976 30216447

[B44] Cabello N. Gandía J. Bertarelli D. C. Watanabe M. Lluís C. Franco R. (2009). Metabotropic glutamate type 5, dopamine D2 and adenosine A2a receptors form higher-order oligomers in living cells. J. Neurochem. 109 (5), 1497–1507. 10.1111/j.1471-4159.2009.06078.x 19344374 PMC3925975

[B45] Calò L. Bruno V. Spinsanti P. Molinari G. Korkhov V. Esposito Z. (2005). Interactions between ephrin-B and metabotropic glutamate 1 receptors in brain tissue and cultured neurons. J. Neurosci. 25 (9), 2245–2254. 10.1523/JNEUROSCI.4956-04.2005 15745950 PMC6726088

[B46] Calsolaro V. Edison P. (2016). Neuroinflammation in Alzheimer’s disease: current evidence and future directions. Alzheimers Dement. 12 (6), 719–732. 10.1016/j.jalz.2016.02.010 27179961

[B47] Canals M. Sexton P. M. Christopoulos A. (2011). Allostery in GPCRs: ‘MWC’ revisited. Trends Biochem. Sci. 36 (12), 663–672. 10.1016/j.tibs.2011.08.005 21920759

[B48] Caraci F. Battaglia G. Bruno V. Bosco P. Carbonaro V. Giuffrida M. L. (2011b). TGF-β1 pathway as a new target for neuroprotection in Alzheimer’s disease. CNS Neurosci. Ther. 17 (4), 237–249. 10.1111/j.1755-5949.2009.00115.x 19925479 PMC6493850

[B49] Caraci F. Molinaro G. Battaglia G. Giuffrida M. L. Riozzi B. Traficante A. (2011a). Targeting group II metabotropic glutamate (mGlu) receptors for the treatment of psychosis associated with Alzheimer’s disease: selective activation of mGlu2 receptors amplifies beta-amyloid toxicity in cultured neurons, whereas dual activation of mGlu2 and mGlu3 receptors is neuroprotective. Mol. Pharmacol. 79 (3), 618–626. 10.1124/mol.110.067488 21159998

[B50] Caraci F. Nicoletti F. Copani A. (2018). Metabotropic glutamate receptors: the potential for therapeutic applications in Alzheimer’s disease. Curr. Opin. Pharmacol. 38, 1–7. 10.1016/j.coph.2017.12.001 29278824

[B51] Cartmell J. Schoepp D. D. (2000). Regulation of neurotransmitter release by metabotropic glutamate receptors. J. Neurochem. 75, 889–907. 10.1046/j.1471-4159.2000.0750889.x 10936169

[B52] Casabona G. Knöpfel T. Kuhn R. Gasparini F. Baumann P. Sortino M. A. (1997). Expression and coupling to polyphosphoinositide hydrolysis of group I metabotropic glutamate receptors in early postnatal and adult rat brain. Eur. J. Neurosci. 9 (1), 12–17. 10.1111/j.1460-9568.1997.tb01348.x 9042564

[B53] Casley C. S. Lakics V. Lee H. G. Broad L. M. Day T. A. Cluett T. (2009). Up-regulation of astrocyte metabotropic glutamate receptor 5 by amyloid-β peptide. Brain Res. 1260, 65–75. 10.1016/j.brainres.2008.12.082 19401173

[B54] Célanire S. Campo B. (2012). Recent advances in the drug discovery of metabotropic glutamate receptor 4 (mGluR4) activators for the treatment of CNS and non-CNS disorders. Expert Opin. Drug Discov. 7 (3), 261–280. 10.1517/17460441.2012.660914 22468956

[B55] Chang W. Pratt S. Chen T. H. Bourguignon L. Shoback D. (2001). Amino acids in the cytoplasmic C terminus of the parathyroid Ca2+-sensing receptor mediate efficient cell-surface expression and phospholipase C activation. J. Biol. Chem. 276 (47), 44129–44136. 10.1074/jbc.M104834200 11535593

[B56] Chang W. Shoback D. (2004). Extracellular Ca2+-sensing receptors→an overview. Cell Calcium 35 (3), 183–196. 10.1016/j.ceca.2003.10.012 15200142

[B57] Chang W. Tu C. Cheng Z. Rodriguez L. Chen T. H. Gassmann M. (2007). Complex formation with the Type B gamma-aminobutyric acid receptor affects the expression and signal transduction of the extracellular calcium-sensing receptor. Studies with HEK-293 cells and neurons. J. Biol. Chem. 282, 25030–25040. 10.1074/jbc.M700924200 17591780

[B58] Chattopadhyay N. Ye C. P. Yamaguchi T. Vassilev P. M. Brown E. M. (1999). Evidence for extracellular calcium-sensing receptor mediated opening of an outward K+ channel in a human astrocytoma cell line (U87). Glia 26 (1), 64–72. 10088673 10.1002/(sici)1098-1136(199903)26:1<64::aid-glia7>3.0.co;2-x

[B59] Chattopadhyay N. (2000). Biochemistry, physiology and pathophysiology of the extracellular calcium-sensing receptor. Int. J. Biochem. Cell Biol. 32 (8), 789–804. 10.1016/S1357-2725(00)00031-5 10940638

[B60] Chen J. H. Ke K. F. Lu J. H. Qiu Y. H. Peng Y. P. (2015). Protection of TGF-β1 against neuroinflammation and neurodegeneration in Aβ1-42-induced Alzheimer’s disease model rats. PLoS One 10 (2), e0116549. 10.1371/journal.pone.0116549 25658940 PMC4319949

[B61] Cheng S. X. Geibe J. P. Hebert S. C. (2004). Extracellular polyamines regulate fluid secretion in rat colonic crypts *via the* extracellular calcium-sensing receptor. Gastroenterology 126, 148–158. 10.1053/j.gastro.2003.10.064 14699496

[B62] Chiarini A. Whitfield J. F. Bonafini C. Chakravarthy B. Armato U. Dal Prà I. (2010). Amyloid-β(25-35), an amyloid-β(1-42) surrogate, and proinflammatory cytokines stimulate VEGF-A secretion by cultured, early passage, normoxic adult human cerebral astrocytes. J. Alzheimer’s Dis. 21, 915–926. 10.3233/JAD-2010-100471 20634577

[B63] Chiarini A. Armato U. Liu D. Dal Prà I. (2016). Calcium-sensing receptors of human neural cells play crucial roles in Alzheimer’s disease. Front. Physiol. 7, 134. 10.3389/fphys.2016.00134 27199760 PMC4844916

[B64] Chiarini A. Armato U. Gardenal E. Gui L. Dal Prà I. (2017a). Amyloid β-exposed human astrocytes overproduce phospho-Tau and overrelease it within exosomes, effects suppressed by calcilytic NPS 2143. Further implications for Alzheimer’s therapy. Front. Neurosci. 11, 217. 10.3389/fnins.2017.00217 28473749 PMC5397492

[B65] Chiarini A. Armato U. Liu D. Dal Prà I. (2017b). Calcium-sensing receptor antagonist NPS 2143 restores amyloid precursor protein physiological non-amyloidogenic processing in Aβ-exposed adult human astrocytes. Sci. Rep. 7 (1), 1277. 10.1038/s41598-017-01215-3 28455519 PMC5430644

[B66] Chiarini A. Armato U. Whitfield J. F. Dal Pra I. (2017c). Targeting human astrocytes’ calcium-sensing receptors for treatment of Alzheimer’s disease. Curr. Pharm. Des. 23 (33), 4990–5000. 10.2174/1381612823666170710162509 28699522

[B67] Choi H. Park H.-H. Lee K. Y. Choi N. Y. Yu H. J. Lee Y. J. (2013). Coenzyme Q10 restores amyloid β-inhibited proliferation of neural stem cells by activating the PI3K pathway. Stem Cells Dev. 22, 2112–2120. 10.1089/scd.2012.0604 23509892

[B68] Christopher J. A. Aves S. J. Bennett K. A. Doré A. S. Errey J. C. Jazayeri A. (2015). Fragment and structure-based drug discovery for a class C GPCR: discovery of the mGlu5 negative allosteric modulator HTL14242 (3-Chloro-5-[6-(5-fluoropyridin-2-yl)pyrimidin-4-yl]benzonitrile). J. Med. Chem. 58 (16), 6653–6664. 10.1021/acs.jmedchem.5b00892 26225459

[B69] Christopoulos A. (2014). Advances in G protein-coupled receptor allostery: from function to structure. Mol. Pharmacol. 86 (5), 463–478. 10.1124/mol.114.094342 25061106

[B70] Chu D. C. Penney J. B. Jr. Young A. B. (1987). Cortical GABAB and GABAA receptors in Alzheimer’s disease: a quantitative autoradiographic study. Neurology 37 (9), 1454–1459. 2819782 10.1212/wnl.37.9.1454

[B71] Ciruela F. Escriche M. Burgueno J. Angulo E. Casado V. Soloviev M. M. (2001). Metabotropic glutamate 1alpha and adenosine A1 receptors assemble into functionally interacting complexes. J. Biol. Chem. 276 (21), 18345–18351. 10.1074/jbc.M006960200 11278325

[B72] Cohen R. M. Rezai-Zadeh K. Weitz T. M. Rentsendorj A. Gate D. Spivak I. (2013). A transgenic Alzheimer rat with plaques, tau pathology, behavioral impairment, oligomeric Aβ, and frank neuronal loss. J. Neurosci. 33, 6245–6256. 10.1523/JNEUROSCI.3672-12.2013 23575824 PMC3720142

[B73] Combs C. K. Johnson D. E. Karlo J. C. Cannady S. B. Landreth G. E. (2000). Inflammatory mechanisms in Alzheimer’s disease: inhibition of b-amyloid-stimulated proinflammatory responses and neurotoxicity by PPAR-gamma agonists. J. Neurosci. 20, 558–567. 10.1523/JNEUROSCI.20-02-00558.2000 10632585 PMC6772401

[B74] Conigrave A. D. Hampson D. R. (2006). Broad-spectrum L-amino acid sensing by class 3 G-protein-coupled receptors. Trends Endocrinol. Metab. 17 (10), 398–407. 10.1016/j.tem.2006.10.012 17085057

[B75] Conigrave A. D. Ward D. T. (2013). Calcium-sensing receptor (CaSR): Pharmacological properties and signaling pathways. Best Pract. Res. Clin. Endocrinol. Metab. 27, 315–331. 10.1016/j.beem.2013.05.010 23856262

[B76] Conley Y. P. Mukherjee A. Kammerer C. DeKosky S. T. Kamboh M. I. Finegold D. N. (2009). Evidence supporting a role for the calcium-sensing receptor in Alzheimer disease. Am. J. Med. Genet. B Neuropsychiatr. Genet. 150B (5), 703–709. 10.1002/ajmg.b.30896 19035514 PMC3062902

[B77] Conn P. J. Pin J. P. (1997). Pharmacology and functions of metabotropic glutamate receptors. Annu. Rev. Pharmacol. Toxicol. 37, 205–237. 10.1146/annurev.pharmtox.37.1.205 9131252

[B78] Conn P. J. Jones C. K. Lindsley C. W. (2009). Subtype-selective allosteric modulators of muscarinic receptors for the treatment of CNS disorders. Trends Pharmacol. Sci. 30 (3), 148–155. 10.1016/j.tips.2008.12.002 19201489 PMC2907736

[B79] Copani A. Bruno V. Battaglia G. Leanza G. Pellitteri R. Russo A. (1995). Activation of metabotropic glutamate receptors protects cultured neurons against apoptosis induced by beta-amyloid peptide. Mol. Pharmacol. 47, 890–897. 7746277

[B80] Copeland C. S. Wall T. M. Sims R. E. Neale S. A. Nisenbaum E. Parri H. R. (2017). Astrocytes modulate thalamic sensory processing *via* mGlu2 receptor activation. Neuropharmacology 121, 100–110. 10.1016/j.neuropharm.2017.04.019 28416443 PMC5480778

[B81] Corrigan F. Vink R. Blumbergs P. C. Masters C. L. Cappai R. van den Heuvel C. (2012). sAPPα rescues deficits in amyloid precursor protein knockout mice following focal traumatic brain injury. J. Neurochem. 122, 208–220. 10.1111/j.1471-4159.2012.07761.x 22519988

[B82] Corti C. Battaglia G. Molinaro G. Riozzi B. Pittaluga A. Corsi M. (2007). The use of knock-out mice unravels distinct roles for mGlu2 and mGlu3 metabotropic glutamate receptors in mechanisms of neurodegeneration/neuroprotection. J. Neurosci. 27 (31), 8297–8308. 10.1523/JNEUROSCI.1889-07.2007 17670976 PMC6673047

[B83] Crimins J. L. Pooler A. Polydoro M. Luebke J. I. Spires-Jones T. L. (2013). The intersection of amyloid β and tau in glutamatergic synaptic dysfunction and collapse in Alzheimer’s disease. Ageing Res. Rev. 12 (3), 757–763. 10.1016/j.arr.2013.03.002 23528367 PMC3735866

[B84] Cummings J. (2017). Lessons learned from Alzheimer disease: clinical trials with negative outcomes. Clin. Trans. Sci. 11, 147–152. 10.1111/cts.12491 PMC5866992 28767185

[B85] Dal Prà I. Chiarini A. Nemeth E. F. Armato U. Whitfield J. F. (2005). Roles of Ca2+ and the Ca2+-sensing receptor (CASR) in the expression of inducible NOS (nitric oxide synthase)-2 and its BH4 (tetrahydrobiopterin)-dependent activation in cytokine-stimulated adult human astrocytes. J. Cell. Biochem. 96 (2), 428–438. 10.1002/jcb.20511 16052472

[B86] Dal Prà I. Whitfileld J. F. Pacchiana R. Bonafini C. Talacchi A. Chakravarthy B. (2011). The amyloid-β42 proxy, amyloid-β(25-35), induces normal human cerebral astrocytes to produce amyloid-β42. J. Alzheimers Dis. 24, 335–347. 10.3233/JAD-2011-101626 21258151

[B87] Dal Prà I. Armato U. Chioffi F. Pacchiana R. Whitfield J. F. Chakravarthy B. (2014a). The Aβ peptides-activated calcium-sensing receptor stimulates the production and secretion of vascular endothelial growth factor-A by normoxic adult human cortical astrocytes. Neuromol. Med. 16 (4), 645–657. 10.1007/s12017-014-8315-9 24948534

[B88] Dal Prà I. Chiarini A. Pacchiana R. Gardenal E. Chakravarthy B. Whitfield J. F. (2014b). Calcium-sensing receptors of human Astrocyte-Neuron Teams: amyloid-β-driven mediators and therapeutic targets of Alzheimer’s Disease. Curr. Neuropharmacol. 12 (4), 353–364. 10.2174/1570159X12666140828214701 25342943 PMC4207075

[B89] Dal Prà I. Chiarini A. Gui L. Chakravarthy B. Pacchiana R. Gardenal E. (2015a). Do astrocytes collaborate with neurons in spreading the “infectious” Aβ and Tau drivers of Alzheimer’s disease? Neuroscientist 21 (1), 9–29. 10.1177/1073858414529828 24740577

[B90] Dal Prà I. Armato U. Chiarini A., (2015b). Specific interactions of calcium-sensing receptors (CaSRs) with soluble amyloid-β peptides→A study using cultured normofunctioning adult human astrocytes. Proceedings of the 2nd International Symposium on the Calcium-sensing Receptor; 2015 March 3–4; San Diego, CA 90–91

[B91] D’Antoni S. Spatuzza M. Bonaccorso C. M. Musumeci S. A. Ciranna L. Nicoletti F. (2014). Dysregulation of group-I metabotropic glutamate (mGlu) receptor mediated signaling in disorders associated with intellectual disability and autism. Neurosci. Biobehav. Rev. 46 Pt 2, 228–241. 10.1016/j.neubiorev.2014.02.003 24548786 PMC5499156

[B92] Davey A. E. Leach K. Valant C. Conigrave A. D. Sexton P. M. Christopolous A. (2012). Positive and negative allosteric modulators promote biased signaling at the calcium-sensing receptor. Endocrinology 153, 4304–4316. 10.1210/en.2012-1449 22210744

[B93] Deng J. Habib A. Obregon D. F. Barger S. W. Giunta B. Wang Y. J.(2015). Soluble amyloid precursor protein-α inhibits tau phosphorylation through modulation of GSK-3β signaling pathway. J. Neurochem. 135, 630–637. 10.1111/jnc.13351 26342176 PMC4624213

[B94] D’Onofrio M. Cuomo L. Battaglia G. Ngomba R. T. Storto M. Kingston A. E. (2001). Neuroprotection mediated by glial group-II metabotropic glutamate receptors requires the activation of the MAP kinase and the phosphatidylinositol-3-kinase pathways. J. Neurochem. 78 (3), 435–445. 10.1046/j.1471-4159.2001.00435.x 11483646

[B95] Di Liberto V. Bonomo A. Frinchi M. Belluardo N. Mudo G. (2010). Group II metabotropic glutamate receptor activation by agonist LY379268 treatment increases the expression of brain derived neurotrophic factor in the mouse brain. Neuroscience 165, 863–873. 10.1016/j.neuroscience.2009.11.012 19909793

[B96] Dinamarca M. C. Raveh A. Schneider A. Fritzius T. Früh S. Rem P. D. (2019). Complex formation of APP with GABA(B) receptors links axonal trafficking to amyloidogenic processing. Nat. Commun. 10 (1), 1331. 10.1038/s41467-019-09164-3 30902970 PMC6430795

[B97] Doré A. S. Okrasa K. Patel J. C. Serrano-Vega M. Bennett K. Cooke R. M. (2014). Structure of class C GPCR metabotropic glutamate receptor 5 transmembrane domain. Nature 511 (7511), 557–562. 10.1038/nature13396 25042998

[B98] Dorr P. Westby M. Dobbs S. Griffin P. Irvine B. Macartney M. (2005). Maraviroc (UK-427,857), a potent, orally bioavailable, and selective small-molecule inhibitor of chemokine receptor CCR5 with broad-spectrum anti-human immunodeficiency virus type 1 activity. Antimicrob. Agents Chemother. 49 (11), 4721–4732. 10.1128/AAC.49.11.4721-4732.2005 16251317 PMC1280117

[B99] Doumazane E. Scholler P. Zwier J. M. Trinquet E. Rondard P. Pin J. P. (2011). A new approach to analyze cell surface protein complexes reveals specific heterodimeric metabotropic glutamate receptors. FASEB J. 25 (1), 66–77. 10.1096/fj.10-163147 20826542

[B100] Durand D. Carniglia L. Caruso C. Lasaga M. (2011). Reduced cAMP, Akt activation and p65-c-Rel dimerization: mechanisms involved in the protective effects of mGluR3 agonists in cultured astrocytes. PLoS One 6 (7), e22235. 10.1371/journal.pone.0022235 21779400 PMC3136520

[B101] Durand D. Carniglia L. Caruso C. Lasaga M. (2013). mGlu3 receptor and astrocytes: partners in neuroprotection. Neuropharmacology 66, 1–11. 10.1016/j.neuropharm.2012.04.009 22564439

[B102] Durand D. Carniglia L. Beauquis J. Caruso C. Saravia F. Lasaga M. (2014). Astroglial mGlu3 receptors promote alpha-secretase-mediated amyloid precursor protein cleavage. Neuropharmacology 79, 180–189. 10.1016/j.neuropharm.2013.11.015 24291464

[B103] Durand D. Carniglia L. Turati J. Ramírez D. Saba J. Caruso C. (2017). Amyloid-beta neurotoxicity and clearance are both regulated by glial group II metabotropic glutamate receptors. Neuropharmacology 123, 274–286. 10.1016/j.neuropharm.2017.05.008 28495373

[B104] Ehlert F. J. (1988). Estimation of the affinities of allosteric ligands using radioligand binding and pharmacological null methods. Mol. Pharmacol. 33 (2), 187–194. 2828914

[B105] El Moustaine D. Granier S. Doumazane E. Scholler P. Rahmeh R. Bron P. (2012). Distinct roles of metabotropic glutamate receptor dimerization in agonist activation and G-protein coupling. Proc. Natl. Acad. Sci. U S A. 109 (40), 16342–16347. 10.1073/pnas.1205838109 22988116 PMC3479612

[B106] Engers D. W. Lindsley C. W. (2013). Allosteric modulation of Class C GPCRs: a novel approach for the treatment of CNS disorders. Drug Discov. Today Technol. 10 (2), e269–e276. 10.1016/j.ddtec.2012.10.007 24050278 PMC3779342

[B107] Espuny-Camacho I. Arranz A. M. Fiers M. Snellinx A. Ando K. Munck S. (2017). Hallmarks of Alzheimer’s disease in stem-cell-derived human neurons transplanted into mouse brain. Neuron 93, 1066–81 e8. 10.1016/j.neuron.2017.02.001 28238547

[B108] Faden A. I. Ivanova S. A. Yakovlev A. G. Mukhin A. G. (1997). Neuroprotective effects of group III mGluR in traumatic neuronal injury. J. Neurotrauma 14, 885–895. 10.1089/neu.1997.14.885 9475370

[B109] Fee C. Banasr M. Sibille E. (2017). Somatostatin-positive gamma-aminobutyric acid interneuron deficits in depression: cortical microcircuit and therapeutic perspectives. Biol. Psychiatry 82 (8), 549–559. 10.1016/j.biopsych.2017.05.024 28697889 PMC5610074

[B110] Ferraguti F. Shigemoto R. (2006). Metabotropic glutamate receptors. Cell Tissue Res. 326, 483–504. 10.1007/s00441-006-0266-5 16847639

[B111] Ferreira S. T. Klein W. L. (2011). The Aβ oligomer hypothesis for synapse failure and memory loss in Alzheimer’s disease. Neurobiol. Learn. Mem. 96 (4), 529–543. 10.1016/j.nlm.2011.08.003 21914486 PMC4390395

[B112] Ferry S. Chatel B. Dodd R. H. Lair C. Gully D. Maffrand J. P. (1997). Effects of divalent cations and of a calcimimetic on adrenocorticotropic hormone release in pituitary tumor cells. Biochem. Biophys. Res. Commun. 238, 866–873. 10.1006/bbrc.1997.7401 9325183

[B113] Foran E. Trotti D. (2009). Glutamate transporters and the excitotoxic path to motor neuron degeneration in amyotrophic lateral sclerosis. Antioxid. Redox Signal. 11, 1587–1602. 10.1089/ars.2009.2444 19413484 PMC2842587

[B114] Fredriksson R. Lagerström M. C. Lundin L. G. Schiöth H. B. (2003). The G-protein-coupled receptors in the human genome form five main families. Phylogenetic analysis, paralogon groups, and fingerprints. Mol. Pharmacol. 63 (6), 1256–1272. 10.1124/mol.63.6.1256 12761335

[B115] Freude K. K. Penjwini M. Davis J. L. LaFerla F. M. Blurton-Jones M. (2011). Soluble amyloid precursor protein induces rapid neural differentiation of human embryonic stem cells. J. Biol. Chem. 286, 24264–24274. 10.1074/jbc.M111.227421 21606494 PMC3129207

[B116] Fribourg M. Moreno J. L. Holloway T. Provasi D. Baki L. Mahajan R. (2011). Decoding the signaling of a GPCR heteromeric complex reveals a unifying mechanism of action of antipsychotic drugs. Cell 147 (5), 1011–1023. 10.1016/j.cell.2011.09.055 22118459 PMC3255795

[B117] Furukawa K. Sopher B. L. Rydel R. E. Begley J. G. Pham D. G. Martin G. M. (1996). Increased activity-regulating and neuroprotective efficacy of α-secretase-derived secreted amyloid precursor protein conferred by a C-terminal heparin-binding domain. J. Neurochem. 67, 1882–1896. 10.1046/j.1471-4159.1996.67051882.x 8863493

[B118] Gabrielczyk T. (2019). Making every cent in testing count. Eur. Biotechnol. (ISSN 2364-2351) 18, 15–19.

[B119] Gafni R. I. Guthrie L. C. Kelly M. H. Brillante B. A. Christie C. M. Reynolds J. C. (2015). Transient increased calcium and calcitriol requirements after discontinuation of human synthetic parathyroid hormone 1-34 (hPTH 1-34) replacement therapy in hypoparathyroidism. J. Bone Miner. Res. 30, 2112–2118. 10.1002/jbmr.2555 25990370 PMC13154038

[B120] Gama L. Wilt S. G. Breitwieser G. E. (2001). Heterodimerization of calcium sensing receptors with metabotropic glutamate receptors in neurons. J. Biol. Chem. 276 (42), 39053–39059. 10.1074/jbc.M105662200 11489900

[B121] Gao L. Jiang T. Yao X. Yu L. Yang X. Li Y. (2017). TREM2 and the progression of Alzheimer’s disease. Curr. Neurovasc. Res. 14 (2), 177–183. 10.2174/1567202614666170404165201 28393702

[B122] Gao R. Penzes P. (2015). Common mechanisms of excitatory and inhibitory imbalance in schizophrenia and autism spectrum disorders. Curr. Mol. Med. 15 (2), 146–167. 10.2174/1566524015666150303003028 25732149 PMC4721588

[B123] Gardenal E. Chiarini A. Armato U. Dal Prà I. Verkhratsky A. Rodríguez J. J. (2017). Increased calcium-sensing receptor immunoreactivity in the hippocampus of a triple transgenic mouse model of Alzheimer’s Disease. Front Neurosci. 11, 81. 10.3389/fnins.2017.00081 28261055 PMC5312420

[B124] Gassmann M. Bettler B. (2012). Regulation of neuronal GABA(B) receptor functions by subunit composition. Nat. Rev. Neurosci. 13 (6), 380–394. 10.1038/nrn3249 22595784

[B125] Geng Y. Bush M. Mosyak L. Wang F. Fan Q. R. (2013). Structural mechanism of ligand activation in human GABA(B) receptor. Nature 504, 254–259. 10.1038/nature12725 24305054 PMC3865065

[B126] Geng Y. Mosyak L. Kurinov I. Zuo H. Sturchler E. Cheng T. C. (2016). Structural mechanism of ligand activation in human calcium-sensing receptor. Elife. 19, 5e13662. 10.7554/eLife.13662 PMC4977154 27434672

[B127] Geurts J. J. G. Wolswijk G. Bö L. Redeker S. Ramkema M. Troost D. (2005). Expression patterns of Group III metabotropic glutamate receptors mGluR4 and mGluR8 in multiple sclerosis lesions. J. Neuroimmunol. 158, 182–190. 10.1016/j.jneuroim.2004.08.012 15589052

[B128] González-Maeso J. Ang R. L. Yuen T. Chan P. Weisstaub N. V. López-Giménez J. F. (2008). Identification of a serotonin/glutamate receptor complex implicated in psychosis. Nature 452 (7183), 93–97. 10.1038/nature06612 18297054 PMC2743172

[B129] Goodman Y. Mattson M. P. (1994). Secreted forms of β-amyloid precursor protein protect hippocampal neurons against amyloid β-peptide-induced oxidative injury. Exp. Neurol. 128, 1–12. 10.1006/exnr.1994.1107 8070512

[B130] Goolam M. A. Ward J. H. Avlani V. A. Leach K. Christopoulos A. Conigrave A. D. (2014). Roles of intraloops-2 and -3 and the proximal C-terminus in signalling pathway selection from the human calcium-sensing receptor. FEBS Lett. 588 (18), 3340–3346. 10.1016/j.febslet.2014.07.022 25080008

[B131] Goudet C. Kniazeff J. Hlavackova V. Malhaire F. Maurel D. Acher F. (2005). Asymmetric functioning of dimeric metabotropic glutamate receptors disclosed by positive allosteric modulators. J. Biol. Chem. 280 (26), 24380–24385. 10.1074/jbc.M502642200 15863499 PMC2557058

[B132] Goudet C. Vilar B. Courtiol T. Deltheil T. Bessiron T. Brabet I. (2012). A novel selective metabotropic glutamate receptor 4 agonist reveals new possibilities for developing subtype selective ligands with therapeutic potential. FASEB J. 26 (4), 1682–1693. 10.1096/fj.11-195941 22223752

[B133] Gouras G. K. Olsson T. T. Hansson O. (2015). β-Amyloid peptides and amyloid plaques in Alzheimer’s disease. Neurotherapeutics 12 (1), 3–11. 10.1007/s13311-014-0313-y 25371168 PMC4322079

[B134] Govindpani K. Calvo-Flores Guzmán B. Vinnakota C. Waldvogel H. J. Faull R. L. Kwakowsky A. (2017). Towards a better understanding of GABAergic remodeling in Alzheimer’s Disease. Int. J. Mol. Sci. 18 (8), E1813. 10.3390/ijms18081813 28825683 PMC5578199

[B135] Gregory K. J. Sexton P. M. Tobin A. B. Christopoulos A. (2012). Stimulus bias provides evidence for conformational constraints in the structure of a G protein-coupled receptor. J. Biol. Chem. 287 (44), 37066–37077. 10.1074/jbc.M112.408534 22965232 PMC3481307

[B136] Gregory K. J. Conn P. J. (2015). Molecular Insights into metabotropic glutamate receptor allosteric modulation. Mol. Pharmacol. 88 (1), 188–202. 10.1124/mol.114.097220 25808929 PMC4468636

[B137] Grolla A. A. Sim J. A. Lim D. Rodriguez J. J. Genazzani A. A. Verkhratsky A. (2013). Amyloid-β and Alzheimer’s disease type pathology differentially affects the calcium signaling toolkit in astrocytes from different brain regions. Cell Death Dis. 4, e623. 10.1038/cddis.2013.145 23661001 PMC3674354

[B138] Grouselle D. Winsky-Sommerer R. David J. P. Delacourte A. Dournaud P. Epelbaum J. (1998). Loss of somatostatin-like immunoreactivity in the frontal cortex of Alzheimer patients carrying the apolipoprotein epsilon 4 allele. Neurosci. Lett. 255 (1), 21–24. /10.1016/S0304-3940(98)00698-3 9839717 10.1016/s0304-3940(98)00698-3

[B139] Gueli M. C. Taibi G. (2013). Alzheimer’s disease: amino acid levels and brain metabolic status. Neurol. Sci. 34 (9), 1575–1579. 10.1007/s10072-013-1289-9 23354600

[B140] Guetg N. Abdel Aziz S. Holbro N. Turecek R. Rose T. Seddik R. (2010). NMDA receptor-dependent GABAB receptor internalization *via* CaMKII phosphorylation of serine 867 in GABAB1. Proc. Natl. Acad. Sci. U S A. 107 (31), 13924–13929. 10.1073/pnas.1000909107 20643921 PMC2922270

[B141] Haberman R. P. Koh M. T. Gallagher M. (2017). Heightened cortical excitability in aged rodents with memory impairment. Neurobiol. Aging 54, 144–151. 10.1016/j.neurobiolaging.2016.12.021 28104309 PMC5401793

[B142] Habib A. Sawmiller D. Tan J. (2016). Restoring soluble amyloid precursor protein-α functions as a potential treatment for Alzheimer’s disease. J. Neurosci. Res. 95, 973–991. 10.1002/jnr.23823 27531392 PMC5296245

[B143] Hall B. Mak E. Cervenka S. Aigbirhio F. I. Rowe J. B. O’Brien J. T. (2017). *In vivo* tau PET imaging in dementia: pathophysiology, radiotracer quantification, and a systematic review of clinical findings. Ageing Res. Rev. 36, 50–63. 10.1016/j.arr.2017.03.002 28315409

[B144] Hamilton A. Esseltine J. L. DeVries R. A. Cregan S. P. Ferguson S. S. (2014). Metabotropic glutamate receptor 5 knockout reduces cognitive impairment and pathogenesis in a mouse model of Alzheimer’s disease. Mol. Brain. 7, 40. 10.1186/1756-6606-7-40 24886239 PMC4050478

[B145] Hannan F. M. Kallay E. Chang W. Brandi M. L. Thakker R. V. (2018). The calcium-sensing receptor in physiology and in calcitropic and noncalcitropic diseases. Nat. Rev. Endocrinol. 15 (1), 33–51. 10.1038/s41574-018-0115-0 30443043 PMC6535143

[B146] Hannan S. Wilkins M. E. Smart T. G. (2012). Sushi domains confer distinct trafficking profiles on GABAB receptors. Proc. Natl. Acad. Sci. U S A. 109 (30), 12171–12176. 10.1073/pnas.1201660109 22778417 PMC3409743

[B147] Hauser A. S. Attwood M. M. Rask-Andersen M. Schiöth H. B. Gloriam D. E. (2017). Trends in GPCR drug discovery: new agents, targets and indications. Nat. Rev. Drug Discov. 16 (12), 829–842. 10.1038/nrd.2017.178 29075003 PMC6882681

[B148] Heidinger V. Manzerra P. Wang X. Q. Strasser U. Yu S. P. Choi D. W. (2002). Metabotropic glutamate receptor 1-induced upregulation of NMDA receptor current: mediation through the Pyk2/Src-family kinase pathway in cortical neurons. J. Neurosci. 22 (13), 5452–5461. 20026565 12097497 10.1523/JNEUROSCI.22-13-05452.2002 PMC6758206

[B149] Hellyer S. D. Albold S. Wang T. Chen A. N. Y. May L. T. Leach K. (2018). Selective class C G protein-coupled receptor modulators are neutral or biased mGlu(5) allosteric ligands. Mol. Pharmacol. 93 (5), 504–514. 10.1124/mol.117.111518 29514854

[B150] Hendy G. N. Guarnieri V. Canaff L. (2009). Calcium-sensing receptor and associated diseases. Prog. Mol. Biol. Transl. Sci. 89, 31–95. 10.1016/S1877-1173(09)89003-0 20374733

[B151] Hendy G. N. Canaff L. Colle D. E. (2013). The CASR gene: alternative splicing and transcriptional control, and calcium-sensing receptor (CaSR) protein: structure and ligand binding sites. Best Pract. Res. Clin. Endocrinol. Metab. 27, 285–301. 10.1016/j.beem.2013.02.009 23856260

[B152] Hick M. Herrmann U. Weyer S. W. Mallm J. P. Tschäpe J. A. Borgers M. (2015). Acute function of secreted amyloid precursor protein fragment APPsα in synaptic plasticity. Acta Neuropath. 129, 21–37. 10.1007/s00401-014-1368-x 25432317

[B153] Hofer A. M. Brown E. M. (2003). Extracellular calcium sensing and signaling. Nat. Rev. Mol. Cell Biol. 4, 530–538. 10.1038/nrm1154 12838336

[B154] Hovelsø N. Sotty F. Montezinho L. Pinheiro P. Herrik K. Mørk A. (2012). Therapeutic potential of metabotropic glutamate receptor modulators. Curr. Neuropharmacol. 10, 12–48. 10.2174/157015912799362805 22942876 PMC3286844

[B155] Hsiao K. Chapman P. Nilsen S. Eckman C. Harigaya Y. Younkin S. (1996). Correlative memory deficits, Abeta elevation, and amyloid plaques in transgenic mice. Science 274, 99–103. 10.1126/science.274.5284.99 8810256

[B156] Hu N. W. Nicoll A. J. Zhang D. Mably A. J. O’Malley T. Purro S. A. (2014). mGlu5 receptors and cellular prion protein mediate amyloid-β-facilitated synaptic long-term depression *in vivo* . Nat. Commun. 5, 3374. 10.1038/ncomms4374 24594908 PMC4354159

[B157] Huang S. Cao J. Jiang M. Labesse G. Liu J. Pin J. P. (2011). Interdomain movements in metabotropic glutamate receptor activation. Proc. Natl. Acad. Sci. U S A. 108, 15480–15485. 10.1073/pnas.1107775108 21896740 PMC3174654

[B158] Huang Y. Breitwieser G. E. (2007). Rescue of calcium-sensing receptor mutants by allosteric modulators reveals a conformational checkpoint in receptor biogenesis. J. Biol. Chem. 282, 9517–9525. 10.1074/jbc.M609045200 17284438

[B159] Huang Y. Mahley R. W. (2014). Apolipoprotein E: structure and function in lipid metabolism, neurobiology, and Alzheimer’s diseases. Neurobiol. Dis. 72 Pt A, 3–12. 10.1016/j.nbd.2014.08.025 25173806 PMC4253862

[B160] Iacovelli L. Bruno V. Salvatore L. Melchiorri D. Gradini R. Caricasole A. (2002). Native group-III metabotropic glutamate receptors are coupled to the mitogen-activated protein kinase/phosphatidylinositol-3-kinase pathways. J. Neurochem. 82, 216–223. 10.1046/j.1471-4159.2002.00929.x 12124422

[B161] Ishimaru H. Ishikawa K. Haga S. Shoji M. Ohe Y. Haga C. (1996). Accumulation of apolipoprotein E and beta-amyloid-like protein in a trace of the hippocampal CA1 pyramidal cell layer after ischaemic delayed neuronal death. Neuroreport 7 (18), 3063–3067. 10.1097/00001756-199611250-00054 9116241

[B162] Ittner L. M. Götz J. (2011). Amyloid-β and tau–a toxic pas de deux in Alzheimer’s disease. Nat. Rev. Neurosci. 12 (2), 65–72. 10.1038/nrn2967 21193853

[B163] Iwakiri M. Mizukami K. Ikonomovic M. D. Ishikawa M. Hidaka S. Abrahamson E. E. (2005). Changes in hippocampal GABABR1 subunit expression in Alzheimer’s patients: association with Braak staging. Acta Neuropathol. 109 (5), 467–474. 10.1007/s00401-005-0985-9 15759131

[B164] Janssens N. Lesage A. S. J. (2001). Glutamate receptor subunit expression in primary neuronal and secondary glial cultures. J. Neurochem. 77, 1457–1474. 10.1046/j.1471-4159.2001.00369.x 11413230

[B165] Jarosz-Griffiths H. H. Noble E. Rushworth J. V. Hooper N. M. (2016). Amyloid-beta receptors: the good, the bad, and the prion protein. J. Biol. Chem. 291, 3174–3183. 10.1074/jbc.R115.702704 26719327 PMC4751366

[B166] Jessen F. Amariglio R. E. van Boxtel M. Breteler M. Ceccaldi M. Chételat G. (2014). Subjective cognitive decline initiative (SCD-I) working group. A conceptual framework for research on subjective cognitivedecline in preclinical Alzheimer’s disease. Alzheimers Dement. 10 (6), 844–852. 10.1016/j.jalz.2014.01.001 24798886 PMC4317324

[B167] Jia Z. Lu Y. Henderson J. Taverna F. Romano C. Abramow-Newerly W. (1998). Selective abolition of the NMDA component of long-term potentiation in mice lacking mGluR5. Learn. Mem. 5 (4-5), 331–343. 10454358 PMC311253

[B168] Jin L.-W. Ninomiya H. Roch J. M. Schubert D. Masliah E. Otero D. A. (1994). Peptides containing the RERMS sequence of amyloid-β/A4 protein precursor bind cell surface and promote neurite extension. J. Neurosci. 14, 5461–5470. 10.1523/JNEUROSCI.14-09-05461.1994 8083748 PMC6577069

[B169] Jo S. Yarishkin O. Hwang Y. J. Chun Y. E. Park M. Woo D. H. (2014). GABA from reactive astrocytes impairs memory in mouse models of Alzheimer’s disease. Nat. Med. 20 (8), 886–896. 10.1038/nm.3639 24973918 PMC8385452

[B170] Jones K. A. Borowsky B. Tamm J. A. Craig D. A. Durkin M. M. Dai M. (1998). GABA(B) receptors function as a heteromeric assembly of the subunits GABA(B)R1 and GABA(B)R2. Nature 396, 674–679. 10.1038/25348 9872315

[B171] Kam T. I. Gwon Y. Jung Y. K. (2014). Amyloid beta receptors responsible for neurotoxicity and cellular defects in Alzheimer’s disease. Cell. Mol. Life Sci. 71, 4803–4813. 10.1007/s00018-014-1706-0 25151011 PMC11113744

[B172] Kammermeier P. J. (2012). Functional and pharmacological characteristics of metabotropic glutamate receptors 2/4 heterodimers. Mol. Pharmacol. 82 (3), 438–447. 10.1124/mol.112.078501 22653971 PMC3422699

[B173] Kang J. Y. Chadchankar J. Vien T. N. Mighdoll M. I. Hyde T. M. Mather R. J. (2017). Deficits in the activity of presynaptic γ-aminobutyric acid type B receptors contribute to altered neuronal excitability in fragile X syndrome. J. Biol. Chem. 292 (16), 6621–6632. 10.1074/jbc.M116.772541 28213518 PMC5399111

[B174] Kaufman A. C. Salazar S. V. Haas L. T. Yang J. Kostylev M. A. Jeng A. T. (2015). Fyn inhibition rescues established memory and synapse loss in Alzheimer mice. Ann. Neurol. 77, 953–971. 10.1002/ana.24394 25707991 PMC4447598

[B175] Kayed R. Lasagna-Reeves C. A. (2013). Molecular mechanisms of amyloid oligomers toxicity. J. Alzheimers Dis. 33 Suppl 1, S67–S78. 10.3233/JAD-2012-129001 22531422

[B176] Keller A. N. Kufareva I. Josephs T. M. Diao J. Mai V. T. Conigrave A. D. (2018). Identification of Global and Ligand-Specific Calcium Sensing Receptor Activation Mechanisms. Mol. Pharmacol. 93 (6), 619–630. 10.1124/mol.118.112086) 29636377 PMC5941188

[B177] Keov P. Sexton P. M. Christopoulos A. (2011). Allosteric modulation of G protein-coupled receptors: a pharmacological perspective. Neuropharmacology 60 (1), 24–35. 10.1016/j.neuropharm.2010.07.010 20637785

[B178] Kessler A. Faure H. Petrel C. Rognan D. Césario M. Ruat M. (2006). N1-Benzoyl-N2-[1-(1-naphthyl)ethyl]-trans-1,2-diaminocyclohexanes: Development of 4-chlorophenylcarboxamide (Calhex 231) as a new calcium-sensing receptor ligand demonstrating potent calcilytic activity. J. Med. Chem. 49, 5119–5128. 10.1021/jm051233+ 16913701

[B179] Khan U. A. Liu L. Provenzano F. A. Berman D. E. Profaci C. P. Sloan R. (2014). Molecular drivers and cortical spread of lateral entorhinal cortex dysfunction in preclinical Alzheimer’s disease. Nat. Neurosci. 17, 304–311. 10.1038/nn.3606 24362760 PMC4044925

[B180] Kim J. Y. Ho H. Kim N. Liu J. Tu C. L. Yenari M. A. (2014). Calcium-sensing receptor (CaSR): a novel target for ischemic neuroprotection. Ann. Clin. Transl. Neurol. 1, 851–866. 10.1002/acn3.118 25540800 PMC4265057

[B181] Kim Y. H. Choi S. H. D’Avanzo C. Hebisch M. Sliwinski C. Bylykbashi E. (2015). A 3D human neural cell culture system for modeling Alzheimer’s disease. Nat. Protoc. 10, 985–1006. 10.1038/nprot.2015.065 26068894 PMC4499058

[B182] Klein W. L. (2013). Synaptotoxic amyloid-β oligomers: a molecular basis for the cause, diagnosis, and treatment of Alzheimer’s disease? J. Alzheimers Dis. 33 Suppl 1, S49–S65. 10.3233/JAD-2012-129039 22785404

[B183] Kniazeff J. Prézeau L. Rondard P. Pin J. P. Goudet C. (2011). Dimers and beyond: the functional puzzles of class C GPCRs. Pharmacol. Ther. 130 (1), 9–25. 10.1016/j.pharmthera.2011.01.006 21256155

[B184] Koole C. Wootten D. Simms J. Valant C. Sridhar R. Woodman O. L. (2010). Allosteric ligands of the glucagon-like peptide 1 receptor (GLP-1R) differentially modulate endogenous and exogenous peptide responses in a pathway-selective manner: implications for drug screening. Mol. Pharmacol. 78, 456–465. 10.1124/mol.110.065664 20547734 PMC3202488

[B185] Kotecha S. A. MacDonald J. F. (2003). Signaling molecules and receptor transduction cascades that regulate NMDA receptor-mediated synaptic transmission. Int. Rev. Neurobiol. 54, 51–106. 12785285 10.1016/s0074-7742(03)54003-x

[B186] Kuhn P. H. Wang H. Dislich B. Colombo A. Zeitschel U. Ellwart J. W. (2010). ADAM10 is the physiologically relevant, constitutive α-secretase of the amyloid precursor protein in primary neurons. EMBO J. 29, 3020–3032. 10.1038/emboj.2010.167 20676056 PMC2944055

[B187] Kumar A. Dhull D. K. Mishra P. S. (2015). Therapeutic potential of mGluR5 targeting in Alzheimer’s disease. Front. Neurosci. 9, 215. 10.3389/fnins.2015.00215 26106290 PMC4460345

[B188] Kunishima N. Shimada Y. Tsuji Y. Sato T. Yamamoto M. Kumasaka T. (2000). Structural basis of glutamate recognition by a dimeric metabotropic glutamate receptor. Nature 407 (6807), 971–977. 10.1038/35039564 11069170

[B189] Lagerström M. C. Schiöth H. B. (2008). Structural diversity of G protein-coupled receptors and significance for drug discovery. Nat. Rev. Drug Discov. 7 (4), 339–357. 10.1038/nrd2518 18382464

[B190] Lan J. Skeberdis V. A. Jover T. Zheng X. Bennett M. V. L. Zukin R. S. (2001). Activation of metabotropic glutamate receptor 1 accelerates NMDA receptor trafficking. J. Neurosci. 21 (16), 6058–6068. 10.1523/JNEUROSCI.21-16-06058.2001 11487629 PMC6763135

[B191] Leach K. Sexton P. M. Christopoulos A. (2007). Allosteric GPCR modulators: taking advantage of permissive receptor pharmacology. Trends Pharmacol. Sci. 28 (8), 382–389. 10.1016/j.tips.2007.06.004 17629965

[B192] Leach K. Conigrave A. D. Sexton P. M. Christopoulos A. (2015). Towards tissue-specific pharmacology: insights from the calcium-sensing receptor as a paradigm for GPCR (patho)physiological bias. Trends Pharmacol. Sci. 36, 215–225. 10.1016/j.tips.2015.02.004 25765207

[B193] Leach K. Gregory K. J. Kufareva I. Khajehali E. Cook A. E. Abagyan R. (2016). Towards a structural understanding of allosteric drugs at the human calcium-sensing receptor. Cell Res. 26 (5), 574–592. 10.1038/cr.2016.36 27002221 PMC4856764

[B194] Lee H. J. Mun H. C. Lewis N. C. Crouch M. F. Culverston E. L. Mason R. S. (2007). Allosteric activation of the extracellular Ca2+-sensing receptor by L-amino acids enhances ERK1/2 phosphorylation. Biochem. J. 404, 141–149. 10.1042/BJ20061826 17212589 PMC1868832

[B195] Lee I. S. Jung K. Kim I. S. Park K. I. (2013). Amyloid-β oligomers regulate the properties of human neural stem cells through GSK-3β signaling. Exp. Mol. Med. 45, e60. 10.1038/emm.2013.125 24232259 PMC3849574

[B196] Lee L. Kosuri P. Arancio O. (2014). Picomolar amyloid-β peptides enhance spontaneous astrocyte calcium transients. J. Alzheimers Dis. 38 (1), 49–62. 10.3233/JAD-130740 23948929 PMC4116306

[B197] Lee M. Schwab C. McGeer P. L. (2011a). Astrocytes are GABAergic cells that modulate microglial activity. Glia 59 (1), 152–165. 10.1002/glia.21087 21046567

[B198] Lee M. McGeer E. G. McGeer P. L. (2011b). Mechanisms of GABA release from human astrocytes. Glia 59 (11), 1600–1611. 10.1002/glia.21202 21748804

[B199] Lehmann K. Steinecke A. Bolz J. (2012). GABA through the ages: regulation of cortical function and plasticity by inhibitory interneurons. Neural Plast. 2012, 892784. 10.1155/2012/892784 22792496 PMC3390141

[B200] Letz S. Rus R. Haag C. Dörr H. G. Schnabel D. Möhlig M. (2010). Novel activating mutations of the calcium-sensing receptor: the calcilytic NPS-2143 mitigates excessive signal transduction of mutant receptors. J. Clin. Endocrinol. Metab. 95, E229–E233. 10.1210/jc.2010-0651 20668040

[B201] Lewczuk P. Kamrowski-Kruck H. Peters O. Heuser I. Jessen F. (2010). Popp, JSoluble amyloid precursor proteins in the cerebrospinal fluid as novel potential biomarkers of Alzheimer’s disease: a multicenter study. Mol. Psychiatry 15, 138–145. 10.1038/mp.2008.84 18663368

[B202] Li Y. Sun H. Chen Z. Xu H. Bu G. Zheng H. (2016). Implications of GABAergic neurotransmission in Alzheimer’s disease. Front. Aging Neurosci. 8, 31. 10.3389/fnagi.2016.00031 26941642 PMC4763334

[B203] Liao J. Schneider A. Datta N. S. McCauley L. K. (2006). Extracellular calcium as a candidate mediator of prostate cancer skeletal metastasis. Cancer Res. 66, 9065–9073. 10.1158/0008-5472.CAN-06-0317 16982748

[B204] Lim D. Iyer A. Ronco V. Grolla A. A. Canonico P. L. Aronica E. (2013). Amyloid beta deregulates astroglial mGluR5-mediated calcium signaling *via* calcineurin and Nf-kB. Glia 61 (7), 1134–1145. 10.1002/glia.22502 23616440

[B205] Losi G. Mariotti L. Carmignoto G. (2014). GABAergic interneuron to astrocyte signaling: a neglected form of cell communication in the brain. Philos. Trans. R. Soc. London B Biol. Sci. 369 (1654), 20130609. 10.1098/rstb.2013.0609 25225102 PMC4173294

[B206] MacInnes N. Messenger M. J. Duty S. (2004). Activation of group III metabotropic glutamate receptors in selected regions of the basal ganglia alleviates akinesia in the reserpine-treated rat. Br. J. Pharmacol. 141 (1), 15–22. 10.1038/sj.bjp.0705566 14597605 PMC1574163

[B207] Magno A. Ward B. K. Rataiczak T. (2011). The calcium-sensing receptor: a molecular perspective. Endocrine. Rev. 32, 3–30. 10.1210/er.2009-0043 20729338

[B208] Maj M. Bruno V. Dragic Z. Yamamoto R. Battaglia G. Inderbitzin W. (2003). (-)-PHCCC, a positive allosteric modulator of mGluR4: characterization, mechanism of action, and neuroprotection. Neuropharmacology 45, 895–906. 10.1016/S0028-3908(03)00271-5 14573382

[B209] Margeta-Mitrovic M. Jan Y. N. Jan L. Y. (2001). Function of GB1 and GB2 subunits in G protein coupling of GABA(B) receptors. Proc. Natl. Acad. Sci. U S A. 98 (25), 14649–14654. 10.1073/pnas.251554498 11724956 PMC64736

[B210] Marshall F. H. Jones K. A. Kaupmann K. Bettler B. (1999). GABAB receptors - the first 7TM heterodimers. Trends Pharmacol. Sci. 20 (10), 396–399. 10.1016/S0165-6147(99)01383-8 10498952

[B211] Mattson M. P. Cheng B. Culwell A. R. Esch F. S. Lieberburg I. Rydel R. E. (1993). Evidence for excitoprotective and intraneuronal calcium-regulating roles for secreted forms of the β-amyloid precursor protein. Neuron 10, 243–254. 10.1016/0896-6273(93)90315-I 8094963

[B212] May L. T. Leach K. Sexton P. M. Christopoulos A. (2007). Allosteric modulation of G protein-coupled receptors. Annu. Rev. Pharmacol. Toxicol. 47, 1–51. 10.1146/annurev.pharmtox.47.120505.105159 17009927

[B213] McKhann G. M. (2011). Changing concepts of Alzheimer disease. JAMA 305 (23), 2458–2459. 10.1001/jama.2011.810 21673298

[B214] Mederos S. González-Arias C. Perea G. (2018). Astrocyte–neuron networks: a multilane highway of signaling for homeostatic brain function. Front. Synaptic Neurosci. 10, 45. 10.3389/fnsyn.2018.00045 30542276 PMC6277918

[B215] Medeiros R. Chabrier M. A. LaFerla F. M. (2013). Elucidating the triggers, progression, and effects of Alzheimer’s disease. J. Alzheimers Dis. 33 Suppl 1, S195–S210. 10.3233/JAD-2012-129009 22635105

[B216] Mennicken F. Maki R. de Souza E. B. Quirion R. (1999). Chemokines and chemokine receptors in the CNS: a possible role in neuroinflammation and patterning. Trends Pharmacol. Sci. 20, 73–78. 10.1016/S0165-6147(99)01308-5 10101968

[B217] Meziane H. Dodart J.-C. Mathis C. Little S. Clemens J. Paul S. M. (1998). Memory enhancing effects of secreted forms of the β-amyloid precursor protein in normal and amnestic mice. Proc. Natl. Acad. Sci. U S A. 95, 12683–12688. 10.1073/pnas.95.21.12683 9770546 PMC22891

[B218] Miedlich S. U. Gama L. Seuwen K. Wolf R. M. Breitwieser G. E. (2004). Homology modeling of the transmembrane domain of the human calcium sensing receptor and localization of an allosteric binding site. J. Biol. Chem. 279, 7254–7263. 10.1074/jbc.M307191200 14660633

[B219] Mihai R. Stevens J. McKinney C. Ibrahim N. B. (2006). Expression of the calcium receptor in human breast cancer→A potential new marker predicting the risk of bone metastases. Eur. J. Surg. Oncol. 32, 511–515. 10.1016/j.ejso.2006.02.009 16564154

[B220] Minakami R. Iida K. Hirakawa N. Sugiyama H. (1995). The expression of two splice variants of metabotropic glutamate receptor subtype 5 in the rat brain and neuronal cells during development. J. Neurochem. 65 (4), 1536–1542. 10.1046/j.1471-4159.1995.65041536.x 7561847

[B221] Morel L. Higashimori H. Tolman M. Yang Y. (2014). VGluT1+ neuronal glutamatergic signaling regulates postnatal developmental maturation of cortical protoplasmic astroglia. J. Neurosci. 34, 10950–10962. 10.1523/JNEUROSCI.1167-14.2014 25122895 PMC4131010

[B222] Motolese M. Mastroiacovo F. Cannella M. Bucci D. Gaglione A. Riozzi B. (2015). Targeting type-2 metabotropic glutamate receptors to protect vulnerable hippocampal neurons against ischemic damage. Mol. Brain 8 (1), 66. 10.1186/s13041-015-0158-2 26496940 PMC4619332

[B223] Moutin E. Raynaud F. Roger J. Pellegrino E. Homburger V. Bertaso F. (2012). Dynamic remodeling of scaffold interactions in dendritic spines controls synaptic excitability. J. Cell Biol. 198 (2), 251–263. 10.1083/jcb.201110101 22801779 PMC3410417

[B224] Muto T. Tsuchiya D. Morikawa K. Jingami H. (2007). Structures of the extracellular regions of the group II/III metabotropic glutamate receptors. Proc. Natl. Acad. Sci. U S A. 104 (10), 3759–3764. 10.1073/pnas.0611577104 17360426 PMC1820657

[B225] Nabers A. Perna L. Lange J. Mons U. Schartner J. Güldenhaupt J. (2018). Amyloid blood biomarker detects Alzheimer’s disease. EMBO Mol. Med. 10 (5), e8763. 10.15252/emmm.201708763 29626112 PMC5938617

[B226] Nakajima Y. Iwakabe H. Akazawa C. Nawa H. Shigemoto R. Mizuno N. (1993). Molecular characterization of a novel retinal metabotropic glutamate receptor mGluR6 with a high agonist selectivity for L-2-amino-4-phosphonobutyrate. J. Biol. Chem. 268, 11868–11873. 8389366

[B227] Nakamura A. Kaneko N. Villemagne V. L. Kato T. Doecke J. Doré V. (2018). High performance plasma amyloid-β biomarkers for Alzheimer’s disease. Nature 554 (7691), 249–254. 10.1038/nature25456 29420472

[B228] Nelson G. Chandrashekar J. Hoon M. A. Feng L. Zhao G. Ryba N. J. P. (2002). An amino-acid taste receptor. Nature 416, 199–202. 10.1038/nature726 11894099

[B229] Nemeth E. F. Delmar E. G. Heaton W. L. Miller M. A. Lambert L. D. Conklin R. L. (2001). Calcilytic compounds: potent and selective Ca2+ receptor antagonists that stimulate secretion of parathyroid hormone. J. Pharmacol. Exp. Ther. 299, 323–331. 11561095

[B230] Nemeth E. F. (2002). The search for calcium receptor antagonists (calcilytics). J. Mol. Endocrinol. 29, 15–21. 10.1677/jme.0.0290015 12200226

[B231] Nemeth E. F. (2004). Calcimimetic and calcilytic drugs: just for parathyroid cells? Cell. Calcium. 35, 283–289. 10.1016/j.ceca.2003.10.020 15200152

[B232] Nemeth E. F. (2013). Allosteric modulators of the extracellular calcium receptor. Drug Discov. Today Technol. 10, e277–e284. 10.1016/j.ddtec.2012.11.002 24050279

[B233] Nemeth E. F. Shoback D. (2013). Calcimimetic and calcilytic drugs for treating bone and mineral-related disorders. Best Pract. Res. Clin. Endocrinol. Metab. 27, 373–384. 10.1016/j.beem.2013.02.008 23856266

[B234] Nemeth E. F. Goodman W. G. (2016). Calcimimetic and calcilytic drugs: feats, flops, and futures. Calcif. Tissue Int. 98, 341–358. 10.1007/s00223-015-0052-z 26319799

[B235] Nesbit M. A. Hannan F. M. Howles S. A. Reed A. A. Cranston T. Thakker C. E. (2013). Mutations in AP2S1 cause familial hypocalciuric hypercalcemia type 3. Nat. Genet. 45 (1), 93–97. 10.1038/ng.2492 23222959 PMC3605788

[B236] Nicoletti F. Iadarola M. J. Wroblewski J. T. Costa E. (1986). Excitatory amino acid recognition sites coupled with inositol phospholipid metabolism: developmental changes and interaction with alpha 1-adrenoceptors. Proc. Natl. Acad. Sci. U S A. 83 (6), 1931–1935. 2869493 10.1073/pnas.83.6.1931 PMC323198

[B237] Nicoletti F. Bruno V. Copani A. Casabona G. Knöpfel T. (1996). Metabotropic glutamate receptors: a new target for the therapy of neurodegenerative disorders? Trends Neurosci. 19, 267–271. 10.1016/S0166-2236(96)20019-0 8799968

[B238] Nicoletti F. Bruno V. Catania M. V. Battaglia G. Copani A. Barbagallo G. (1999). Group-I metabotropic glutamate receptors: hypotheses to explain their dual role in neurotoxicity and neuroprotection. Neuropharmacology 38 (10), 1477–1484. 10.1016/S0028-3908(99)00102-1 10530809

[B239] Niswender C. M. Johnson K. A. Weaver C. D. Jones C. K. Xiang Z. Luo Q. (2008). Discovery, characterization, and antiparkinsonian effect of novel positive allosteric modulators of metabotropic glutamate receptor 4. Mol. Pharmacol. 74, 1345–1358. 10.1124/mol.108.049551 18664603 PMC2574552

[B240] Niswender C. M. Conn P. J. (2010). Metabotropic glutamate receptors: physiology, pharmacology, and disease. Annu. Rev. Pharmacol. Toxicol. 50, 295–322. 10.1146/annurev.pharmtox.011008.145533 20055706 PMC2904507

[B241] Noh J. S. Pak H. J. Shin Y. J. Riew T. R. Park J. H. Moon Y. W. (2015). Differential expression of the calcium-sensing receptor in the ischemic and border zones after transient focal cerebral ischemia in rats. J. Chem. Neuroanat. 66-67, 40–51. 10.1016/j.jchemneu.2015.05.001 26013410

[B242] Nuriel T. Angulo S. L. Khan U. Ashok A. Chen Q. Figueroa H. Y. (2017). Neuronal hyperactivity due to loss of inhibitory tone in APOE4 mice lacking Alzheimer’s disease-like pathology. Nat. Commun. 8 (1), 1464. 10.1038/s41467-017-01444-0 29133888 PMC5684208

[B243] Nygaard H. B. (2018). Targeting Fyn kinase in Alzheimer’s disease. Biol. Psychiatry 83 (4), 369–376. 10.1016/j.biopsych.2017.06.004 28709498 PMC5729051

[B244] Obregon D. Hou H. Deng J. Hou H. Deng J. Giunta B., (2012). Soluble amyloid precursor protein-α modulates β-secretase activity and amyloid-β generation. Nat. Commun. 3, 777. 10.1038/ncomms1781 22491325 PMC3520614

[B245] Oddo S. Caccamo A. Shepherd J. D. Murphy M. P. Golde T. E. Kayed R. (2003). Triple-transgenic model of Alzheimer’s disease with plaques and tangles: Intracellular A-β and synaptic dysfunction. Neuron 39, 409–421. 10.1016/S0896-6273(03)00434-3 12895417

[B246] Oh S. J. Lee C. J. (2017). Distribution and function of the Bestrophin-1 (Best1) channel in the brain. Exp. Neurobiol. 26 (3), 113–121. 10.5607/en.2017.26.3.113 28680296 PMC5491579

[B247] Ohsawa I. Takamura C. Kohsaka S. (1997). The amino-terminal region of amyloid precursor protein is responsible for neurite outgrowth in rat neocortical explant culture. Biochem. Biophys. Res. Commun. 236, 59–65. 10.1006/bbrc.1997.6903 9223426

[B248] Orts-Del’Immagine A. Pugh J. R. (2018). Activity-dependent plasticity of presynaptic GABA(B) receptors at parallel fiber synapses. Synapse 72 (5), e22027. 10.1002/syn.22027 29360168 PMC6003616

[B249] Pacheco-Quinto J. Eckman C. B. Eckman E. A. (2016). Major amyloid-β-degrading enzymes, endothelin-converting enzyme-2 and neprilysin, are expressed by distinct populations of GABAergic interneurons in hippocampus and neocortex. Neurobiol. Aging 48, 83–92. 10.1016/j.neurobiolaging.2016.08.011 27644077 PMC5159282

[B250] Palmqvist S. Janelidze S. Stomrud E. Zetterberg H. Karl J. Zink K. (2019). Performance of fully automated plasma assays as screening tests for Alzheimer disease–related β-amyloid status. JAMA Neurol. 10.1001/jamaneurol.2019.1632 epub ahead of print PMC6593637 31233127

[B251] Palucha A. Pilc A. (2007). Metabotropic glutamate receptor ligands as possible anxiolytic and antidepressant drugs. Pharmacol. Ther. 115, 116–147. 10.1016/j.pharmthera.2007.04.007 17582504

[B252] Park J. H. Ju Y. H. Choi J. W. Song H. J. Jang B. K. Woo J. (2019). Newly developed reversible MAO-B inhibitor circumvents the shortcomings of irreversible inhibitors in Alzheimer’s disease. Sci. Adv. 5 (3), eaav0316. 10.1126/sciadv.aav0316 30906861 PMC6426469

[B253] Park S. Y. Mun H. C. Eom Y. S. Baek H. L. Jung T. S. Kim C. H. (2013). Identification and characterization of D410E, a novel mutation in the loop 3 domain of CaSR, in autosomal dominant hypocalcemia and a therapeutic approach using a novel calcilytic, AXT914. Clin. Endocrinol. (Oxf). 78, 687–693. 10.1111/cen.12056 23009664

[B254] Parmentier-Batteur S. Hutson P. H. Menzel K. Uslaner J. M. Mattson B. A. O’Brien J. A. (2014). Mechanism based neurotoxicity of mGlu5 positive allosteric modulators–development challenges for a promising novel antipsychotic target. Neuropharmacology 82, 161–173. 10.1016/j.neuropharm.2012.12.003 23291536

[B255] Parnot C. Kobilka B. (2004). Toward understanding GPCR dimers. Nat. Struct. Mol. Biol. 11 (8), 691–692. 10.1038/nsmb0804-691 15280880

[B256] Pei J. J. Braak E. Braak H. Grundke-Iqbal I. Iqbal K. Winblad B. (1999). Distribution of active glycogen synthase kinase 3beta (GSK-3beta) in brains staged for Alzheimer disease neurofibrillary changes. J. Neuropathol. Exp. Neurol. 58 (9), 1010–1019. 10.1097/00005072-199909000-00011 10499443

[B257] Pelkey K. A. Yuan X. Lavezzari G. Roche K. W. McBain C. J. (2007). mGluR7 undergoes rapid internalization in response to activation by the allosteric agonist AMN082. Neuropharmacology 52, 108–117. 10.1016/j.neuropharm.2006.07.020 16914173

[B258] Perroy J. Prezeau L. De Waard M. Shigemoto R. Bockaert J. Fagni L. (2000). Selective blockade of P/Q-type calcium channels by the metabotropic glutamate receptor type 7 involves a phospholipase C pathway in neurons. J. Neurosci. 20, 7896–7904. 10.1523/JNEUROSCI.20-21-07896.2000 11050109 PMC6772734

[B259] Peters-Libeu C. Campagna J. Mitsumori M. Poksay K. S. Spilman P. Sabogal A. (2015). sAβPPα is a potent endogenous inhibitor of BACE1. J. Alzheimers Dis. 47, 545–555. 10.3233/JAD-150282 26401691

[B260] Petrel C. Kessler A. Masliah F. Dauban P. Dodd R. H. Rognan D. (2003). Modeling and mutagenesis of the binding site of Calhex 231, a novel negative allosteric modulator of the extracellular Ca(2+)-sensing receptor. J. Biol. Chem. 278, 49487–49494. 10.1074/jbc.M308010200 14506236

[B261] Petrel C. Kessler A. Dauban P. Dodd R. H. Rognan D. Ruat M. (2004). Positive and negative allosteric modulators of the Ca2+-sensing receptor interact within overlapping but not identical binding sites in the transmembrane domain. J. Biol. Chem. 279, 18990–18997. 10.1074/jbc.M400724200 14976203

[B262] Petroff O. A. (2002). GABA and glutamate in the human brain. Neuroscientist 8 (6), 562–573. 10.1177/1073858402238515 12467378

[B263] Phillips T. Makoff A. Murrison E. Mimmack M. Waldvogel H. Faull R. (1998). Immunohistochemical localisation of mGluR7 protein in the rodent and human cerebellar cortex using subtype specific antibodies. Mol. Brain Res. 57, 132–141. /10.1016/S0169-328X(98)00081-3 9630572 10.1016/s0169-328x(98)00081-3

[B264] Pidasheva S. Grant M. Canaff L. Ercan O. Kumar U. Hendy G. N. (2006). Calcium-sensing receptor dimerizes in the endoplasmic reticulum: biochemical and biophysical characterization of CASR mutants retained intracellularly. Hum. Mol. Gen. 15, 2200–2209. 10.1093/hmg/ddl145 16740594

[B265] Pisani A. Gubellini P. Bonsi P. Conquet F. Picconi B. Centonze D. (2001). Metabotropic glutamate receptor 5 mediates the potentiation of N-methyl-D-aspartate responses in medium spiny striatal neurons. Neuroscience 106 (3), 579–587. 10.1016/S0306-4522(01)00297-4 11591458

[B266] Podlisny M. B. Tolan D. R. Selkoe D. J. (1991). Homology of the amyloid beta protein precursor in monkey and human supports a primate model for beta amyloidosis in Alzheimer’s disease. Am. J. Pathol. 138, 1423–1435. 1905108 PMC1886384

[B267] Price M. R. Baillie G. L. Thomas A. Stevenson L. A. Easson M. Goodwin R. (2005). Allosteric modulation of the cannabinoid CB1 receptor. Mol. Pharmacol. 68 (5), 1484–1495. 10.1124/mol.105.016162 16113085

[B268] Purgert C. A. Izumi Y. Jong Y. J. Kumar V. Zorumski C. F. O’Malley K. L. (2014). Intracellular mGluR5 can mediate synaptic plasticity in the hippocampus. J. Neurosci. 34 (13), 4589–4598. 10.1523/JNEUROSCI.3451-13.2014 24672004 PMC3965784

[B269] Qiu W. Q. Ferreira A. Miller C. Koo E. H. Selkoe D. J. (1995). Cell-surface β-amyloid precursor protein stimulates neurite outgrowth of hippocampal neurons in an isoform-dependent manner. J. Neurosci. 15, 2157–2167. 10.1523/JNEUROSCI.15-03-02157.1995 7891158 PMC6578166

[B270] Quinn S. J. Ye C. P. Diaz R. Kifor O. Bai M. Vassilev P. (1997). The Ca^2+^-sensing receptor: a target for polyamines. Am J Physiol. 273, C1315–C1323. 9357776 10.1152/ajpcell.1997.273.4.C1315

[B271] Quiroz Y. T. Budson A. E. Celone K. Ruiz A. Newmark R. Castrillón G. (2010). Hippocampal hyperactivation in presymptomatic familial Alzheimer’s disease. Ann. Neurol. 68 (6), 865–875. 10.1002/ana.22105 21194156 PMC3175143

[B272] Rammes G. Hasenjäger A. Sroka-Saidi K. Deussing J. M. Parsons C. G. (2011). Therapeutic significance of NR2B-containing NMDA receptors and mGluR5 metabotropic glutamate receptors in mediating the synaptotoxic effects of β-amyloid oligomers on long-term potentiation (LTP) in murine hippocampal slices. Neuropharmacology 60 (6), 982–990. 10.1016/j.neuropharm.2011.01.051 21310164

[B273] Ransohoff R. M. (2018). All animal models of neurodegeneration are wrong. Are they also useful? J. Exp. Med 215, 2955–2958. 10.1084/jem.20182042 30459159 PMC6279414

[B274] Renner M. Lacor P. N. Velasco P. T. Xu J. Contractor A. Klein W. L. (2010). Deleterious effects of amyloid beta oligomers acting as an extracellular scaffold for mGluR5. Neuron 66 (5), 739–754. 10.1016/j.neuron.2010.04.029 20547131 PMC3111138

[B275] Riccardi D. Kemp P. J. (2012). The calcium-sensing receptor beyond extracellular calcium homeostasis: conception, development, adult physiology, and disease. Annu. Rev. Physyiol. 74, 271–297. 10.1146/annurev-physiol-020911-153318 22017175

[B276] Riccardi D. Brennan S. C. Chang W. (2013). The extracellular calcium-sensing receptor, CaSR, in fetal development. Best Pract. Res. Clin. Endocrinol. Metab. 27, 443–453. 10.1016/j.beem.2013.02.010 23856271 PMC4462341

[B277] Rice H. C. de Malmazet D. Schreurs A. Frere S. Van Molle I. Volkov A. N. (2019). Secreted amyloid-β precursor protein functions as a GABABR1a ligand to modulate synaptic transmission. Science 363 (6423), eaao4827. 10.1126/science.aao4827 30630900 PMC6366617

[B278] Ring S. Weyer S. W. Kilian S. B. Waldron E. Pietrzik C. U. Filippov M. A. (2007). The secreted β-amyloid precursor protein ectodomain APPsα is sufficient to rescue the anatomical, behavioral, and electrophysiological abnormalities of APP deficient mice. J. Neurosci. 27, 7817–7826. 10.1523/JNEUROSCI.1026-07 17634375 PMC6672885

[B279] Ritzén A. Mathiesen J. M. Thomsen C. (2005). Molecular pharmacology and therapeutic prospects of metabotropic glutamate receptor allosteric modulators. Basic Clin. Pharmacol. Toxicol. 97 (4), 202–213. 10.1111/j.1742-7843.2005.pto_156.x 16176554

[B280] Roch J.-M. Masliah E. Roch-Levecq A.-C. Sundsmo M. P. Otero D. A. Veinbergs I. (1994). Increase of synaptic density and memory retention by a peptide representing the trophic domain of the amyloid-β/A4 protein precursor. Proc. Natl. Acad. Sci. U S A. 91, 7450–7454. 10.1073/pnas.91.16.7450 8052602 PMC44418

[B281] Romano C. van den Pol A. N. O’Malley K. L. (1996). Enhanced early developmental expression of the metabotropic glutamate receptor mGluR5 in rat brain: protein, mRNA splice variants, and regional distribution. J. Comp. Neurol. 367 (3), 403–412. 10.1002/(SICI)1096-9861(19960408)367:3<403::AID-CNE6>3.0.CO;2-9 8698900

[B282] Rondard P. Liu J. Huang S. Malhaire F. Vol C. Pinault A. (2006). Coupling of agonist binding to effector domain activation in metabotropic glutamate-like receptors. J. Biol. Chem. 281 (34), 24653–24661. 10.1074/jbc.M602277200 16787923

[B283] Rondard P. Goudet C. Kniazeff J. Pin J. P. Prézeau L. (2011). The complexity of their activation mechanism opens new possibilities for the modulation of mGlu and GABAB class C G protein-coupled receptors. Neuropharmacology 60 (1), 82–92. 10.1016/j.neuropharm.2010.08.009 20713070

[B284] Rosenbaum D. M. Rasmussen S. G. Kobilka B. K. (2009). The structure and function of G-protein-coupled receptors. Nature 459 (7245), 356–363. 10.1038/nature08144 19458711 PMC3967846

[B285] Rosenbaum D. M. Zhang C. Lyons J. A. Holl R. Aragao D. Arlow D. H. (2011). Structure and function of an irreversible agonist-β(2) adrenoceptor complex. Nature 469, 236–240. 10.1038/nature09665 21228876 PMC3074335

[B286] Roy U. Stute L. Höfling C. Hartlage-Rübsamen M. Matysik J. Roβner S. (2018). Sex- and age-specific modulation of brain GABA levels in a mouse model of Alzheimer’s disease. Neurobiol. Aging 62, 168–179. 10.1016/j.neurobiolaging.2017.10.015 29154037

[B287] Ruat M. Traiffort E. (2013). Roles of the calcium sensing receptor in the central nervous system. Best Pract. Res. Clin. Endocrinol. Metab. 27, 429–442. 10.1016/j.beem.2013.03.001 23856270

[B288] Sabelhaus C. F. Schröder U. H. Breder J. Henrich-Noack P. Reymann K. G. (2000). Neuroprotection against hypoxic/hypoglycaemic injury after the insult by the group III metabotropic glutamate receptor agonist (R, S)-4-phosphonophenylglycine. Br. J. Pharmacol. 131, 655–658. 10.1038/sj.bjp.0703646 11030711 PMC1572399

[B289] Saidak Z. Brazier M. Kamel S. Mentaverri R. (2009). Agonists and modulators of the calcium-sensing receptor and their therapeutic applications. Mol. Pharmacol. 76, 1131–1144. 10.1124/mol.109.058784 19779033

[B290] Sasaguri H. Nilsson P. Hashimoto S. Nagata K. Saito T. De Strooper B. (2017). APP mouse models for Alzheimer’s disease pre-clinical studies. EMBO J. 36, 2473–2487. 10.15252/embj.201797397 28768718 PMC5579350

[B291] Shigemoto R. Kinoshita A. Wada E. Nomura S. Ohishi H. Takada M. (1997). Differential presynaptic localization of metabotropic glutamate receptor subtypes in the rat hippocampus. J. Neurosci. 17, 7503–7522. 10.1523/JNEUROSCI.17-19-07503.1997 9295396 PMC6573434

[B292] Schoepp D. D. Johnson B. G. (1989). Inhibition of excitatory amino acid-stimulated phosphoinositide hydrolysis in the neonatal rat hippocampus by 2-amino-3-phosphonopropionate. J. Neurochem. 53 (6), 1865–1870. 2572680 10.1111/j.1471-4159.1989.tb09254.x

[B293] Schoepp D. D. (2001). Unveiling the functions of presynaptic metabotropic glutamate receptors in the central nervous system. J. Pharmacol. Exp. Ther. 299, 12–20. 11561058

[B294] Selkoe D. J. (2008a). Biochemistry and molecular biology of amyloid beta-protein and the mechanism of Alzheimer’s disease. Handb. Clin. Neurol. 89, 245–260. 10.1016/S0072-9752(07)01223-7 18631749

[B295] Selkoe D. J. (2008b). Soluble oligomers of the amyloid beta-protein impair synaptic plasticity and behavior. Behav. Brain Res. 192 (1), 106–113. 10.1016/j.bbr.2008.02.016 18359102 PMC2601528

[B296] Sengmany K. Singh J. Stewart G. D. Conn P. J. Christopoulos A. Gregory K. J. (2017). Biased allosteric agonism and modulation of metabotropic glutamate receptor 5: implications for optimizing preclinical neuroscience drug discovery. Neuropharmacology 115, 60–72. 10.1016/j.neuropharm.2016.07.001 27392634 PMC5217481

[B297] Sevastyanova T. N. Kammermeier P. J. (2014). Cooperative signaling between homodimers of metabotropic glutamate receptors 1 and 5. Mol. Pharmacol. 86 (5), 492–504. 10.1124/mol.114.093468 25113912 PMC4201138

[B298] Shah A. Silverstein P. S. Singh D. P. Kumar A. (2012). Involvement of metabotropic glutamate receptor 5, AKT/PI3K signaling and NF-κB pathway in methamphetamine-mediated increase in IL-6 and IL-8 expression in astrocytes. J. Neuroinflammation 9, 52. 10.1186/1742-2094-9-52 22420994 PMC3338363

[B299] Shrivastava A. N. Kowalewski J. M. Renner M. Bousset L. Koulakoff A. Melki R. (2013). β-amyloid and ATP-induced diffusional trapping of astrocyte and neuronal metabotropic glutamate type-5 receptors. Glia 61 (10), 1673–1686. 10.1002/glia.22548 23922225

[B300] Skeberdis V. A. Lan J. Opitz T. Zheng X. Bennett M. V. Zukin R. S. (2001). mGluR1-mediated potentiation of NMDA receptors involves a rise in intracellular calcium and activation of protein kinase C. Neuropharmacology 40 (7), 856–865. 10.1016/S0028-3908(01)00005-3 11378156

[B301] Silve C. Petrel C. Leroy C. Bruel H. Mallet E. Rognan D. (2005). Delineating a Ca^2+^ binding pocket within the venus flytrap module of the human calcium-sensing receptor. J. Biol. Chem. 280 (45), 37917–37923. 10.1074/jbc.M506263200 16147994

[B302] Slack S. Battaglia A. Cibert-Goton V. Gavazzi I. (2008). EphrinB2 induces tyrosine phosphorylation of NR2B *via* Src-family kinases during inflammatory hyperalgesia. Neuroscience 156 (1), 175–183. 10.1016/j.neuroscience.2008.07.023 18694808 PMC2568875

[B303] Smith-Swintosky V. L. Pettigrew L. C. Craddock S. D. Culwell A. R. Rydel R. E. Mattson M. P. (1994). Secreted forms of β-amyloid precursor protein protect against ischemic brain injury. J. Neurochem. 63, 781–784. /101046/j.1471-4159199463020781.x 8035204 10.1046/j.1471-4159.1994.63020781.x

[B304] Sokol D. K. Maloney B. Long J. M. Ray B. Lahiri D. K. (2011). Autism, Alzheimer disease, and fragile X: APP, FMRP, and mGluR5 are molecular links. Neurology 76 (15), 1344–1352. 10.1212/WNL.0b013e3182166dc7 21482951 PMC3090060

[B305] Spampinato S. F. Molinaro G. Merlo S. Iacovelli L. Caraci F. Battaglia G. (2012). Estrogen receptors and type 1 metabotropic glutamate receptors are interdependent in protecting cortical neurons against β-amyloid toxicity. Mol. Pharmacol. 81 (1), 12–20. 10.1124/mol.111.074021 21984253

[B306] Stachowicz K. Chojnacka-Wójcik E. Kłak K. Pilc A. (2007). Anxiolytic-like effect of group III mGlu receptor antagonist is serotonin-dependent. Neuropharmacology 52 (2), 306–312. 10.1016/j.neuropharm.2006.08.002 17020774

[B307] Stansley B. J. Conn P. J. (2019). Neuropharmacological insight from allosteric modulation of mGlu receptors. Trends Pharmacol. Sci. 40 (4), 240–252. 10.1016/j.tips.2019.02.006 30824180 PMC6445545

[B308] Stein T. D. Johnson J. A. (2003). Genetic programming by the proteolytic fragments of the amyloid precursor protein: somewhere between confusion and clarity. Rev. Neurosci. 14, 317–342. 14640319 10.1515/revneuro.2003.14.4.317

[B309] Sun W. McConnell E. Pare J. F. Xu Q. Chen M. Peng W. (2013). Glutamate-dependent neuroglial calcium signaling differs between young and adult brain. Science 339, 197–200. 10.1126/science.1226740 23307741 PMC3569008

[B310] Takahashi H. Brasnjevic I. Rutten B. P. Van Der Kolk N. Perl D. P. Bouras C. (2010). Hippocampal interneuron loss in an APP/PS1 double mutant mouse and in Alzheimer’s disease. Brain Struct. Funct. 214 (2-3), 145–160. 10.1007/s00429-010-0242-4 20213270 PMC3038332

[B311] Talantova M. Sanz-Blasco S. Zhang X. Xia P. Akhtar M. W. Okamoto S. (2013). Aβ induces astrocytic glutamate release, extrasynaptic NMDA receptor activation, and synaptic loss. Proc. Natl. Acad. Sci. U.S.A. 110 (27), E2518–E2527. 10.1073/pnas.1306832110 23776240 PMC3704025

[B312] Tang B. L. (2019). Amyloid precursor protein (APP) and GABAergic neurotransmission. Cells 8, 550. 10.3390/cells8060550 PMC6627941 31174368

[B313] Tharmalingam S. Wu C. Hampson D. R. (2016). The calcium-sensing receptor and integrins modulate cerebellar granule cell precursor differentiation and migration. Dev. Neurobiol. 76 (4), 375–389. 10.1002/dneu.22321 26138678

[B314] Terunuma M. Vargas K. J. Wilkins M. E. Ramírez O. A. Jaureguiberry-Bravo M. Pangalos N. (2010). Prolonged activation of NMDA receptors promotes dephosphorylation and alters post endocytic sorting of GABAB receptors. Proc. Natl. Acad. Sci. U S A. 107 (31), 13918–13923. 10.1073/pnas.1000853107 20643948 PMC2922229

[B315] Thompson S. E. Ayman G. Woodhall G. L. Jones R. S. (2006–2007). Depression of glutamate and GABA release by presynaptic GABAB receptors in the entorhinal cortex in normal and chronically epileptic rats. Neurosignals 15 (4), 202–215. 10.1159/000098515 PMC2504722 17215590

[B316] Thomsen A. R. Hvidtfeldt M. Bräuner-Osborne H. (2012). Biased agonism of the calcium-sensing receptor. Cell Calcium 51 (2), 107–116. 10.1016/j.ceca.2011.11.009 22192592

[B317] Tichauer J. E. von Bernhardi R. (2012). Transforming growth factor-β stimulates β-amyloid uptake by microglia through Smad3-dependent mechanisms. J. Neurosci. Res. 90 (10), 1970–1980. 10.1002/jnr.23082 22715062

[B318] Tsuchiya D. Kunishima N. Kamiya N. Jingami H. Morikawa K. (2002). Structural views of the ligand-binding cores of a metabotropic glutamate receptor complexed with an antagonist and both glutamate and Gd3^+^ . Proc. Natl. Acad. Sci. U S A. 99 (5), 2660–2665. 10.1073/pnas.052708599 11867751 PMC122404

[B319] Tu J. C. Xiao B. Naisbitt S. Yuan J. P. Petralia R. S. Brakeman P. (1999). Coupling of mGluR/Homer and PSD-95 complexes by the Shank family of postsynaptic density proteins. Neuron 23 (3), 583–592. 10.1016/S0896-6273(00)80810-7 10433269

[B320] Um J. W. Kaufman A. C. Kostylev M. Heiss J. K. Stagi M. Takahashi H. (2013). Metabotropic glutamate receptor 5 is a coreceptor for Alzheimer Aβ oligomer bound to cellular prion protein. Neuron 79 (5), 887–902. 10.1016/j.neuron.2013.06.036 24012003 PMC3768018

[B321] Unger M. S. Marschallinger J. Kaindl J. Höfling C. Rossner S. Heneka M. T. (2016). Early changes in hippocampal neurogenesis in transgenic mouse models for Alzheimer’s disease. Mol. Neurobiol. 53, 5796–5806. 10.1007/s12035-016-0018-9 27544234 PMC5012146

[B322] Urwyler S. (2011). Allosteric modulation of family C G-protein-coupled receptors: from molecular insights to therapeutic perspectives. Pharmacol. Rev. 63 (1), 59–126. 10.1124/pr.109.002501 21228259

[B323] Valant C. Felder C. C. Sexton P. M. Christopoulos A. (2012). Probe dependence in the allosteric modulation of a G protein-coupled receptor: implications for detection and validation of allosteric ligand effects. Mol. Pharmacol. 81, 41–52. 10.1124/mol.111.074872 21989256

[B324] Vanzulli I. Butt A. M. (2015). mGluR5 protect astrocytes from ischemic damage in postnatal CNS white matter. Cell Calcium 58 (5), 423–430. 10.1016/j.ceca.2015.06.010 26189008 PMC4634333

[B325] Verkhratsky A. Nedergaard M. (2018). Physiology of astroglia. Physiol. Rev. 98 (1), 239–389. 10.1152/physrev.00042.2016 29351512 PMC6050349

[B326] Vernon A. Palmer S. Datla K. Zbarsky V. Croucher M. Dexter D. (2005). Neuroprotective effects of metabotropic glutamate receptor ligands in a 6-hydroxydopamine rodent model of Parkinson’s disease. Eur. J. Neurosci. 22, 1799–1806. 10.1111/j.1460-9568.2005.04362.x 16197521

[B327] Villette V. Dutar P. (2017). GABAergic microcircuits in Alzheimer’s disease models. Curr. Alzheimer Res. 14 (1), 30–39. 10.2174/1567205013666160819125757 27539596

[B328] Ward B. K. Magno A. L. Walsh J. P. Ratajczak T. (2012). The role of the calcium-sensing receptor in human disease. Clin. Biochem. 45, 943–953. 10.1016/j.clinbiochem.2012.03.034 22503956

[B329] White J. H. Wise A. Main M. J. Green A. Fraser N. J. Disney G. H. (1998). Heterodimerization is required for the formation of a functional GABA(B) receptor. Nature 396 (6712), 679–682. 10.1038/25354 9872316

[B330] White E. McKenna J. Cavanaugh A. Breitwieser G. E. (2009). Pharmacochaperone-mediated rescue of calcium-sensing receptor loss-of-function mutants. Mol. Endocrinol. 23, 1115–1123. 10.1210/me.2009-0041 19389809 PMC2703600

[B331] Widler L. Altmann E. Beerli R. Breitenstein W. Bouhelal R. Buhl T. (2010). 1-Alkyl-4-phenyl-6-alkoxy-1H-quinazolin-2-ones: a novel series of potent calcium-sensing receptor antagonists. J. Med. Chem. 53 (5), 2250–2263. 10.1021/jm901811v 20158186

[B332] Widler L. (2011). Calcilytics: antagonists of the calcium-sensing receptor for the treatment of osteoporosis. Future Med. Chem. 3, 535–547. 10.4155/fmc.11.17 21526895

[B333] Williams C. J. Dexter D. T. (2014). Neuroprotective and symptomatic effects of targeting group III mGlu receptors in neurodegenerative disease. J Neurochem. 129 (1), 4–20. 10.1111/jnc.12608 24224472

[B334] Wootten D. Christopoulos A. Sexton P. M. (2013). Emerging paradigms in GPCR allostery: implications for drug discovery. Nat. Rev. Drug Discov. 12 (8), 630–644. 10.1038/nrd4052 23903222

[B335] Wu H. Wang C. Gregory K. J. Han G. W. Cho H. P. Xia Y. (2014). Structure of a class C GPCR metabotropic glutamate receptor 1 bound to an allosteric modulator. Science 344 (6179), 58–64. 10.1126/science.1249489 24603153 PMC3991565

[B336] Xiong M. Jones O. D. Peppercorn K. Ohline S. M. Tate W. P. Abraham W. C. (2016). Secreted amyloid precursor protein-α can restore novel object location memory and hippocampal LTP in aged rats. Neurobiol. Learn. Mem. S1074-7427 (16), 30144–30147. 10.1016/j.nlm.2016.08.002 27521248

[B337] Xu M. Y. Wong A. H. C. (2018). GABAergic inhibitory neurons as therapeutic targets for cognitive impairment in schizophrenia. Acta Pharmacol. Sin. 39 (5), 733–753. 10.1038/aps.2017.172 29565038 PMC5943898

[B338] Yamamura A. Guo Q. Yamamura H. Zimnicka A. M. Pohl N. M. Smith K. A. (2012). Enhanced Ca2+-sensing receptor function in idiopathic pulmonary arterial hypertension. Circ. Res. 111, 469–481. 10.1161/CIRCRESAHA.112.266361 22730443 PMC3695473

[B339] Yamamura A. Ohara N. Tsukamoto K. (2015). Inhibition of excessive cell proliferation by calcilytics in idiopathic arterial hypertension. PLoS One 10, e0138384. 10.1371/journal.pone.0138384 26375676 PMC4574199

[B340] Yao H. H. Ding J. H. Zhou F. Wang F. Hu L. F. Sun T. (2005). Enhancement of glutamate uptake mediates the neuroprotection exerted by activating group II or III metabotropic glutamate receptors on astrocytes. J. Neurochem. 92, 948–961. 10.1111/j.1471-4159.2004.02937.x 15686497

[B341] Yano S. Brown E. M. Chattopadhyay N. (2004). Calcium-sensing receptor in the brain. Cell Calcium 35 (3), 257–264. 10.1016/j.ceca.2003.10.008 15200149

[B342] Yarova P. L. Stewart A. L. Sathish V. Britt R. D. Thompson M. A. Plowe A. P. (2015). Calcium-sensing receptor antagonists abrogate airway hyper-responsiveness and inflammation in allergic asthma. Sci. Transl Med. 7, 284ra60. 10.1126/scitranslmed.aaa0282 PMC4725057 25904744

[B343] Ye C. Ho-Pao C. L. Kanazirska M. Quinn S. Rogers K. Seidman C. E. (1997). Amyloid-beta proteins activate Ca(2+)-permeable channels through calcium-sensing receptors. J. Neurosci. Res. 47, 547–554. 10.1002/(SICI)1097-4547(19970301)47:5<547::AID-JNR10>3.0.CO;2-V 9067864

[B344] Yin S. Noetzel M. J. Johnson K. A. Zamorano R. Jalan-Sakrikar N. Gregory K. J. (2014). Selective actions of novel allosteric modulators reveal functional heteromers of metabotropic glutamate receptors in the CNS. J. Neurosci. 34, 79–94. 10.1523/JNEUROSCI.1129-13.2014 24381270 PMC3866496

[B345] Zhang C. Miller C. L. Brown E. M. Yang J. J. (2015a). The calcium sensing receptor: from calcium sensing to signaling. Sci. China Life Sci. 58 (1), 14–27. 10.1007/s11427-014-4779-y 25576451

[B346] Zhang D. Zhao Q. Wu B. (2015b). Structural studies of G protein-coupled receptors. Mol. Cells 38 (10), 836–842. 10.14348/molcells.2015.0263 26467290 PMC4625064

[B347] Zhang Z. Sun S. Quinn S. J. Brown E. M. Bai M. (2001). The extracellular calcium-sensing receptor dimerizes through multiple types of intermolecular interactions. J. Biol. Chem. 276, 5316–5322. 10.1074/jbc.M005958200 11069904

[B348] Zhou F. Yao H. H. Wu J. Y. Yang Y. J. Ding J. H. Zhang J. (2006). Activation of Group II/III metabotropic glutamate receptors attenuates LPS-induced astroglial neurotoxicity *via* promoting glutamate uptake. J. Neurosci. Res. 84, 268–277. 10.1002/jnr.20897 16752416

[B349] Zhuang X. Northup J. K. Ray K. (2012). Large putative PEST-like sequence motif at the carboxyl tail of human calcium receptor directs lysosomal degradation and regulates cell surface receptor level. J. Biol. Chem. 287 (6), 4165–4176. 10.1074/jbc.M111.271528 22158862 PMC3281744

